# Recent Advances in Xenes Based FET for Biosensing Applications

**DOI:** 10.1002/advs.202500752

**Published:** 2025-05-14

**Authors:** Huide Wang, Chen Wang, Yule Zhang, Ziqian Wang, Yihan Zhu, Yun Wang, Xiangqian Hong, Han Zhang, Ning Fan, Meng Qiu

**Affiliations:** ^1^ State Key Laboratory of Radio frequency Heterogeneous integration International Collaborative Laboratory of 2D Materials for Optoelectronics Science and Technology Institute for Advanced Study in Nuclear Energy and Safety Interdisciplinary Center of High Magnetic Field Physics of Shenzhen University College of Physics and Optoelectronic Engineering Shenzhen University Shenzhen 518060 China; ^2^ Key Laboratory of Marine Chemistry Theory and Technology (Ministry of Education) College of Chemistry and Chemical Engineering Ocean University of China Qingdao 266100 China; ^3^ Shenzhen Eye Hospital Shenzhen Eye Institute Jinan University Shenzhen 518040 China

**Keywords:** 2D materials, biosensing, fabrication techniques, FET, Xenes

## Abstract

In recent years, monoelemental 2D materials (Xenes) such as graphene, graphdiyne, silicene, phosphorene, and tellurene, have gained significant traction in biosensing applications. Owing to their ultra‐thin layered structure, exceptionally high specific surface area, unique surface electronic properties, excellent mechanical strength, flexibility, and other distinctive features, Xenes are recognized for their potential as materials with low detection limits, high speed, and exceptional flexibility in biosensing applications. In this review, the unique properties of Xenes, their synthesis, and recent theoretical and experimental advances in applications related to biosensing, including DNA/RNA biosensors, protein biosensors, small molecule biosensors, cell, and ion biosensors are comprehensively summarized. Finally, the challenges and prospects of this emerging field are discussed.

## Graphical abstract

Xenes (monoelemental 2D materials like graphene, phosphorene, tellurene) enable ultrasensitive FET (Field‐Effect Transistor) biosensors due to their tunable bandgap, high carrier mobility, and large surface area. Advances in synthesis (CVD, MBE) and functionalization allow detection of DNA/RNA (via CRISPR‐Cas systems), proteins, small molecules, and ions with femtomolar limits. Challenges include environmental stability, scalable fabrication, and integration into portable systems. Future focuses on heterostructures, surface engineering, and clinical validation for real‐world diagnostics.

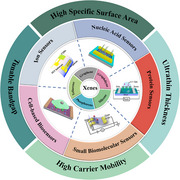

## 1 Introduction

Biosensing technology plays a crucial role in disease diagnosis and therapeutic monitoring by detecting biomarkers with high sensitivity and excellent precision.^[^ ^1^, ^2^ ^]^ For instance, monitoring blood glucose levels can closely track fluctuations in diabetic patients,^[^ ^3^, ^4^ ^]^ while detecting cholesterol levels can help assess an individual's risk of cardiovascular diseases.^[^ ^5^, ^6^, ^7^ ^]^ The core metrics, including accuracy, response speed, cost‐effectiveness, sensitivity, and portability, jointly determine its effectiveness.^[^ ^8^, ^9^, ^10^ ^]^ Common detection methods for biomarkers include electrochemical luminescence sensors,^[^ ^11^, ^12^, ^13^ ^]^ field‐effect transistor (FET) sensors,^[^ ^14^, ^15^, ^16^, ^17^, ^18^, ^19^ ^]^ enzyme‐linked immunosorbent assay (ELISA),^[^ ^20^, ^21^ ^]^ polymerase chain reaction (PCR),^[^ ^22^, ^23^ ^]^ mass spectrometry,^[^ ^24^, ^25^ ^]^ and fluorescence spectroscopy.^[^ ^26^, ^27^ ^]^ FET‐based sensors have demonstrated significant advantages such as high integration, inherent signal amplification, low power consumption, and ultra‐short response times.^[^ ^10^, ^28^ ^]^ In 1980, researchers pioneered the innovative concept of combining FETs with enzymes, demonstrating the successful immobilization of penicillinase on a silicon‐doped FET channel. This early system achieved penicillin detection with a remarkable detection limit of 0.2 mM, a response time of 25 s, and a sensing area of only 0.5 mm^2^.^[^ ^29^ ^]^ However, ​these first‐generation FET biosensors relied on conventional semiconductor channels (e.g., silicon, tin oxide) that exhibited three critical limitations: 1) low sensitivity, 2) stringent detection condition requirements, and 3) drain current escalation upon miniaturization. These critical limitations are expected to be overcome through the strategic selection and engineering of FET channel materials,^[^ ^30^, ^31^ ^]^ which fundamentally redefines the semiconductor channel material as a pivotal design parameter requiring strategic consideration in FET biosensor engineering.

Recently, FET biosensor devices based on 2D materials are being continuously developed.^[^ ^32^, ^33^, ^34^ ^]^ Due to their unparalleled sensitivity, versatility, and potential for miniaturization, 2D materials FET offer a wide range of applications in biosensing, allowing the detection of various biomolecules.^[^ ^35^, ^36^ ^]^ 2D materials can overcome the destructive increase in drain current caused by miniaturization, primarily due to their atomic thickness enabling near‐ideal electrostatic gate control and intrinsic high carrier mobility. Even when channel lengths are scaled below 5 nm, these materials suppress drain current surges induced by short‐channel effects. Furthermore, their self‐passivated surfaces significantly reduce interface trap density, minimizing carrier scattering and enabling linear enhancement of drain current density with scaling, unlike traditional silicon‐based materials which exhibit uncontrollable performance degradation. However, conventional 2D material systems exhibit significant limitations. The sulfur vacancy‐induced Fermi‐level pinning in transition metal dichalcogenides (TMDs) severely disrupts the threshold voltage stability of FETs,^[^ ^37^ ^]^ while their limited surface functionalization site density directly constrains probe molecule modification efficiency. The metallic conductivity of MXenes results in excessively low current on/off ratios, reducing sensitivity to subtle biomolecular charges.^[^ ^38^ ^]^ Notably, the hygroscopic surfaces of MXenes significantly amplify errors caused by nonspecific adsorption in physiological environments.^[^ ^39^ ^]^ Additionally, the strong interfacial phonon scattering effects common to both TMDs and MXenes lead to carrier mobility degradation, ultimately limiting biomolecular detection capabilities.^[^ ^40^ ^]^ In contrast, monoelemental 2D materials (Xenes) such as graphene, graphdiyne, phosphorene, borophene, tellurene, and antimonene, which possess high specific surface area, ultrathin thickness, high carrier mobility, tunable bandgap, and in‐plane anisotropy, have garnered significant interest.^[^ ^41^, ^42^, ^43^, ^44^ ^]^ Their high specific surface area and ultrathin thickness indicate extremely high loading capacity and minimal signal noise, leading to a theoretically ultralow limit of detection (LOD).^[^ ^41^, ^44^, ^45^, ^46^ ^]^ Theoretical calculations and simulation studies provide a microscopic‐level explanation for the superior properties of Xenes.^[^ ^47^, ^48^, ^49^ ^]^ Density functional theory (DFT) calculations reveal that the electronic structures of Xenes (such as G and BP) possess highly tunable bandgap characteristics, whose surface charge distribution can be regulated through layer number, thereby optimizing electrostatic interactions with biomolecules.^[^ ^50^ ^]^ The adaptive surface of Xenes allows it to be modified with different biomarkers, which aids in the identification of certain biomolecules and enhances the sensitivity and analytical range of biosensors.^[^ ^10^, ^45^, ^51^, ^52^ ^]^ Biomolecules modified on Xenes act as gates, altering the channel's carrier concentration, which in turn changes the FET's conductivity and allows for the detection of biomolecules.^[^ ^53^, ^54^ ^]^ The sensing performance of Xenes FETs is affected by several factors: the type of Xenes, the properties of the dielectric layer, the degree of surface functionalization, and the contact resistance. By optimizing these parameters, the LOD, sensitivity, stability, selectivity, and detection range of Xenes FET sensors can be improved. Leveraging the unique properties of Xenes, FETs based on Xenes such as graphene,^[^ ^55^ ^]^ graphdiyne^[^ ^56^ ^]^ and phosphorene,^[^ ^57^ ^]^ have been reported to have great potential in detecting DNA,^[^ ^58^, ^59^ ^]^ RNA,^[^ ^60^ ^]^ proteins,^[^ ^61^, ^62^ ^]^ small biomolecules,^[^ ^63^ ^]^ cells,^[^ ^64^ ^]^ and ions.^[^ ^65^ ^]^ The sensing mechanisms of Xenes FET biosensors can be categorized into electrostatic gating effects and charge transfer effects.^[^ ^28^ ^]^ In addition, composite structures based on Xenes can also serve as channel materials for FET biosensors, such as graphene/oxide‐graphene van der Waals heterostructure FET biosensors.^[^ ^62^ ^]^

Herein, the developments in FET biosensors based on Xenes in recent years are reviewed. First, the Xenes FET biosensing platform is introduced from four aspects, including basic concepts, classifications, working principles and performance indicators. Then, the structure, characteristics, and fabrication methods of Xenes are discussed in detail. Subsequently, the unique advantages of Xenes in FET biosensing are discussed. Further, the latest research progresses of Xenes FET biosensors used in the detection of DNA/RNA, proteins, small biomolecules, and cell detection are summarized. Finally, the challenges and prospects for the development of Xenes‐based FET biosensors are discussed.

## 2 Xenes FET Biosensing Platform

Xenes FET biosensors integrate a FET platform with specific recognition elements to achieve high‐sensitivity detection of external disturbances. In this section, the basic concepts, classification, working principles, and performance evaluation criteria of Xenes FET sensors are summarized.

### 2.1 Basic Concepts of Xenes FET biosensors

FET is a conventional three‐terminal electronic device that consists of four primary components: source (S), gate (G), drain (D), and the semiconductor channel situated between the source and drain.^[^ ^66^ ^]^ The Xenes FET shares a similar structure with the conventional FET, with the notable distinction that the channel material situated between the source and drain comprises Xenes. The FET biosensor represents an efficient and sensitive platform for electrochemical detection.^[^ ^67^ ^]^ Xenes FET employed as biosensors leverages the inherent structure of the transistor to integrate biological receptors within the gate, channel, or other components. Due to the high input impedance, low power consumption, and excellent high‐frequency characteristics of Xenes FET, they have a broad application prospect in fields such as communication, computers, and consumer electronics, especially playing a significant role in the design of high‐speed and low‐power integrated circuits.

Three different types of curves are usually used to describe FET biosensors: the transfer characteristic curve (*I*_DS_‐*V*_GS_), the output characteristic curve (*I*_DS_‐*V*_DS_), and the transient response curve (*I*_DS_‐*T*).

**Figure 1:**
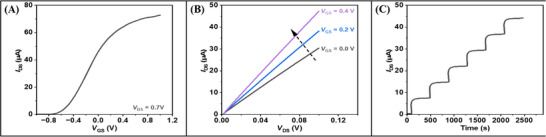
Characteristic curve of Xenes FET biosensor. A) Typical transfer characteristics of Xenes FET biosensors. B) Typical output characteristics of Xenes FET biosensors. C) Typical real‐time response curves of Xenes FET biosensors.

*I*_DS_‐*V*_GS_. Under a constant drain voltage, the transfer characteristic curve of a FET is obtained by scanning the gate voltage to observe the changes in drain current. The parameters of transistor doping type,^[^ ^68^ ^]^ carrier migration, and Dirac voltage^[^ ^59^ ^]^ can be derived from the curve. For an enhancement‐mode FET, when *V*_GS_ is below the threshold voltage (*V*_th_), I_DS_ is almost zero. When the gate voltage exceeds *V*_th_, *I*_DS_ increases with the increase of gate voltage (**Figure** **1**A). For a depletion‐mode FET, even when *V*_GS_ is zero, there is a certain *I*_DS_, and changing *V*_GS_ can further adjust the size of *I*_DS_. The transfer characteristic curve can reflect the switching characteristics and amplification capability of the FET.

*I*_DS_‐*V*_DS_. The output characteristic curve illustrates the relationship between the drain voltage and the drain current under a specified grid voltage.^[^ ^10^ ^]^ The output characteristic curve provides a direct representation of the contact state between the metal electrode of a device and its channel material. An ideal, linear output characteristic curve signifies ohmic contact between the metal electrode and the semiconductor material (Figure 1B). Conversely, a non‐linear curve indicates the presence of a contact barrier at the metal‐semiconductor contact interface, referred to as Schottky contact, which impedes efficient charge transport within the device channel.^[^ ^69^ ^]^

*I*_DS_‐*T*. The real‐time impact of external interference on channel current is reflected in the transient response curve.^[^ ^10^ ^]^ The drain current will gradually vary with the addition of biological samples of different concentrations (Figure 1C).^[^ ^70^, ^71^ ^]^

### 2.2 Classifications of Xenes FET Biosensors

**Figure 2:**
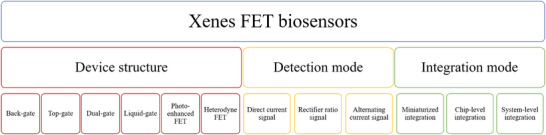
Representative Classification of Xenes FET biosensors.

The Xenes FET biosensors can be classified based on three perspectives of device structure, detection mode, and integration mode, as shown in **Figure** **2**.

#### 2.2.1 Classification Based on Device Structure

**Figure 3:**
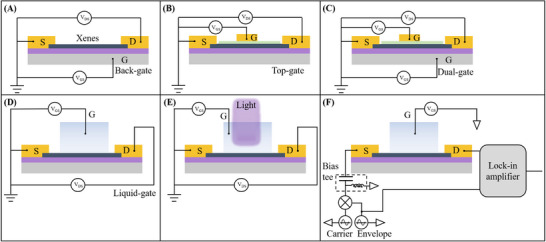
Classification of Xenes FET from the point of view of device structure. A) Schematic diagram of back‐gate FET. B) Schematic diagram of top‐gate FET. C) Schematic diagram of dual‐gate FET. D) Schematic diagram of liquid‐gate FET. E) Schematic diagram of photo‐enhanced FET. F) Schematic diagram of heterodyne FET.

Xenes FET can be categorized into four main types based on their gate configurations: back‐gate (**Figure** **3**A), top‐gate (Figure 3B), dual‐gate (Figure 3C), and liquid‐gated FET (Figure 3D). FET with different gate structures exhibit distinct performance characteristics and application scenarios.^[^ ^10^ ^]^ Back‐gated FET offer simple fabrication and low cost but suffer from weak control capability and low integration density, making them suitable for fundamental material research and flexible electronics.^[^ ^72^ ^]^ Top‐gated FET, with their gate structures tightly integrated near the channel, achieve strong electric field coupling and high integration density, and are preferred for high‐performance integrated circuits and high‐frequency devices, despite challenges in fabrication complexity.^[^ ^73^ ^]^ Dual‐gated FET enable dynamic control of carrier concentration and type through independent dual‐gate regulation, offering advantages in low‐power logic devices and reconfigurable circuits, though their design complexity and larger footprint hinder high‐density integration.^[^ ^74^ ^]^ Liquid‐gated FET utilize liquid electrolyte interfaces to achieve ultrahigh sensitivity and biocompatibility, and are widely used in biosensing and electrochemical analysis, despite limitations in stability and encapsulation.^[^ ^75^ ^]^ Ultimately, selecting the appropriate structure requires balancing control capability, fabrication complexity, and application‐specific demands. Given that biological detection processes are commonly carried out in solution environments, liquid‐gated FET have garnered significant attention due to their extensive applications in the field of biosensing. In addition to the widely used liquid‐gated FET structures, some researchers have begun to explore the application of light exposure in the field of FET sensing. For instance, Wei et al. developed photo‐enhanced FETs (Figure 3E) for the detection of small molecules, indicating the potential application prospects of Xenes FETs in the field of optoelectronics sensing. Furthermore, Kulkarni et al. focused on the biosensing applications of heterodyne FET, investigating the beat frequency response between molecular dipoles and FET by applying high‐frequency alternating current signals to the FET, providing a new perspective for FET biosensing technology (Figure 3F).

#### 2.2.2 Classification Based on Detection Modes

FET can be categorized into three detection modes: direct current signal FET, rectifier ratio signal FET, and alternating current signal FET. In the current research domain, Xenes FET biosensors primarily employ direct current FET technology. This technique monitors changes in detection signals such as drain current or Dirac voltage, thereby reflecting variations in the concentration of biomolecules. Rectifier ratio signal FET, on the other hand, obtain detection signals by calculating the ratio of positive bias voltage to negative bias voltage. This rectifier ratio signal not only serves as a signal response to monitor changes in biomolecule concentration but also effectively reduces the interference of external environmental factors on the signal as well as signal differences among different devices. Additionally, alternating current signal FET utilize mixed current as the response signal for the detection of biomolecules. Studies have shown that this detection method can surpass the Debye length limitation, achieving high‐sensitivity detection of biomolecules, with significantly improved detection sensitivity compared to direct current signal FET. These three detection modes, each targeting different signal characteristics, provide a diversified selection for Xenes FET biosensing.

#### 2.2.3 Classification Based on Integration Mode

**Figure 4:**
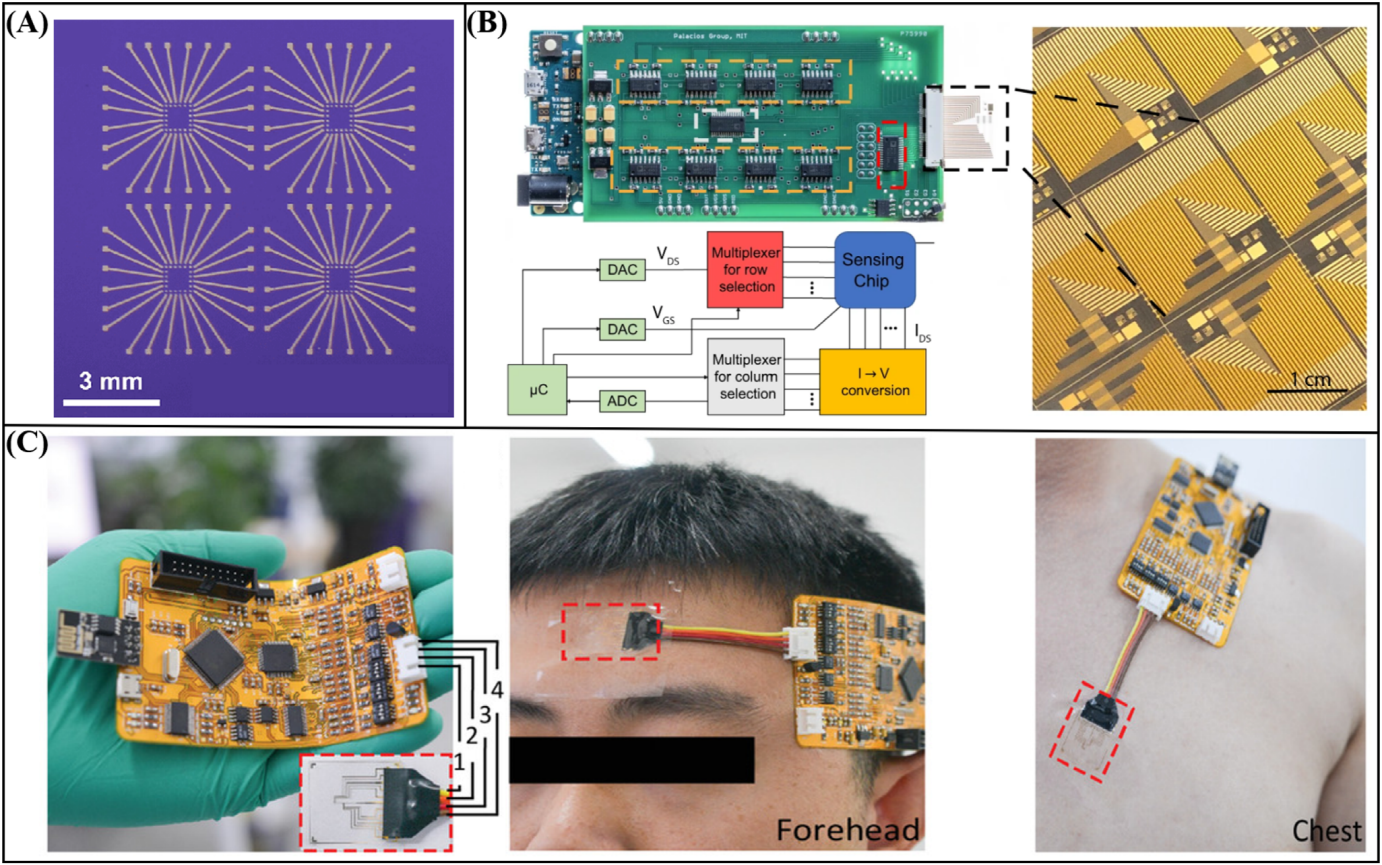
Integration mode of Xenes FET. A) Schematic diagram of miniaturized integration.^[^ ^76^ ^]^ Copyright 2020, Science China Press and Springer‐Verlag. B) Schematic diagram of chip‐level integration.^[^ ^77^ ^]^ Copyright 2022, Springer Nature. C) Schematic diagram of system ‐level integration.^[^ ^78^ ^]^ Copyright 2021, Wiley‐VCH.

In the classification of integration methods for Xenes FET biosensors, they can be categorized based on the complexity and scale of integration into three levels: miniaturized integration, chip‐level integration, and system‐level integration. Miniaturized integration involves the integration of multiple FET sensors on a single substrate, thereby enabling the detection of various biomolecules on the same platform, although each sensor remains essentially a single‐point detection device (**Figure** **4**A). Chip‐level integration further develops this concept by integrating a large number of sensing units, custom high‐speed readout electronics, and machine learning inference modules on a single chip, thereby achieving rapid, portable, and reliable measurements (Figure 4B). System‐level integration represents the advanced stage of development for Xenes FET biosensors, aiming to develop highly integrated applications such as wearable sensor systems or biomimetic sensor systems. Currently, research has been conducted to integrate system‐level Xenes FET biosensors with wearable devices for measuring physiological biomarkers, facilitating continuous health monitoring (Figure 4C).

### 2.3 Working Principles of Xenes FET Biosensors

The interaction between the target molecule and Xenes modifies the channel's conductivity. This change in conductivity is the fundamental principle behind the detection capabilities of Xenes FET sensors.^[^ ^10^, ^79^, ^80^ ^]^ By correlating the change in channel current with the concentration of the target biomolecule, the sensor allows for quantitative detection of biomolecules. Interactions between Xenes and the target molecules include electrostatic adsorption,^[^ ^81^ ^]^ specific biomolecular binding,^[^ ^59^, ^82^ ^]^ and chemical reactions^[^ ^70^, ^83^ ^]^ occurring at the sensing interface.

**Figure 5:**
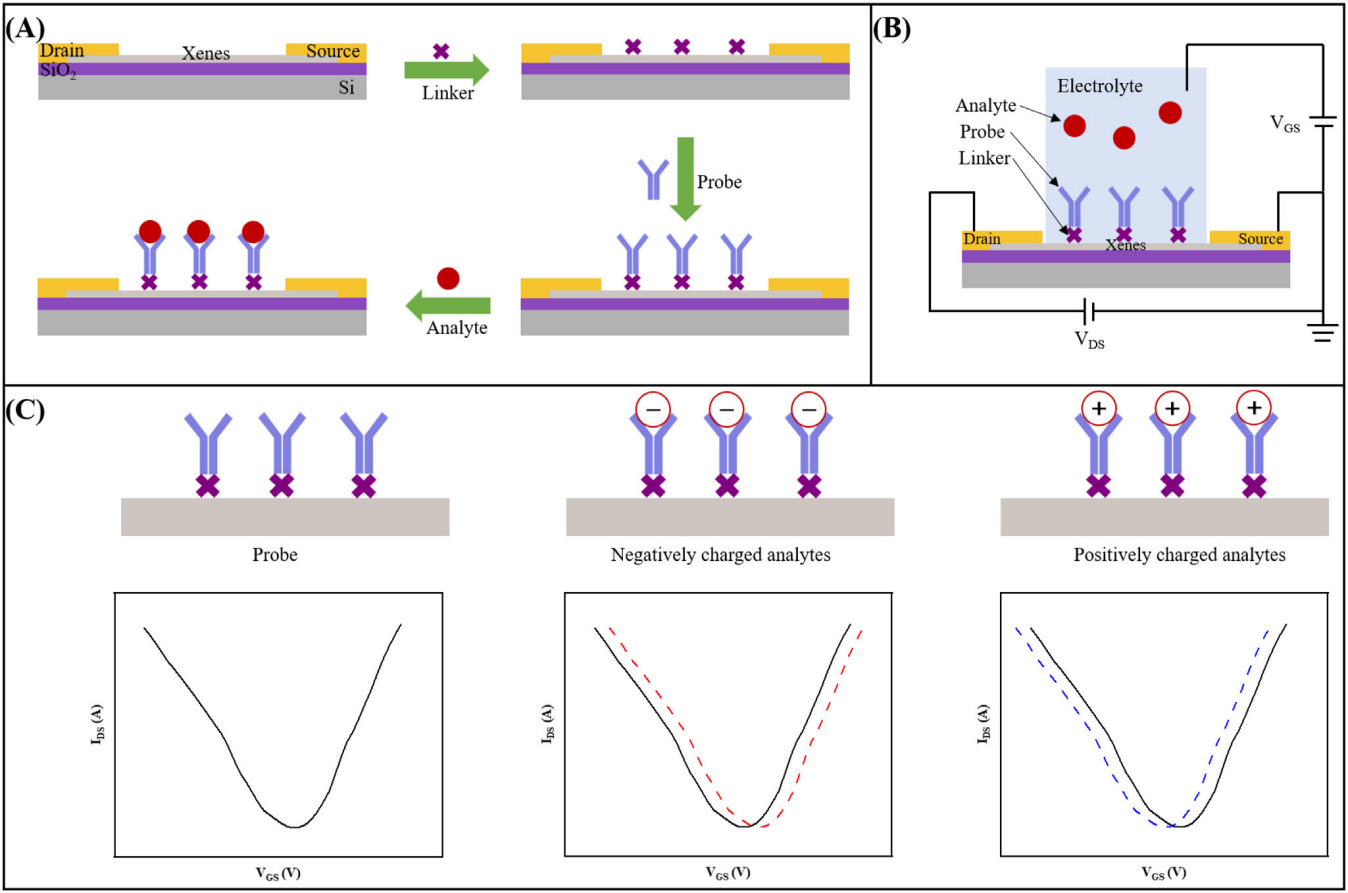
The working principle of the Xenes FET biosensor. A) Schematic diagram of the functionalization process of the Xenes FET biosensor. B) Liquid‐gate Xenes FET biosensor schematic. C) Sensing principle of the Xenes FET biosensor: The shift in the transfer characteristic curve occurs during the detection of various charged biomolecules.

**Figure** **5**A depicts the construction process of the Xenes FET biosensor. First, the linker is modified on the Xenes surface. Biological probes with specific properties (e.g., DNA probes,^[^ ^84^, ^85^ ^]^ antibodies^[^ ^61^, ^82^ ^]^) are subsequently attached to the linker, thereby immobilizing them on the Xenes surface. Finally, the modified Xenes FET biosensor is employed in detecting specific biomolecules, capturing them and generating measurable signals. Figure 5B graphically depicts a liquid‐gate Xenes FET biosensor with a reference electrode supplying a gate voltage through an electrolyte solution. Figure 5C illustrates the evolving transfer characteristic curve of the Xenes FET biosensor when detecting differently charged biomolecules. Specifically, when the biomolecule is negatively charged, there is a decrease in the Dirac voltage, resulting in a leftward shift of the curve, indicative of an n‐doping effect in the electrolyte solution.^[^ ^86^ ^]^ Conversely, when the biomolecule is positively charged, the curve exhibits an opposite trend.

The sensing performance of Xenes FET biosensors critically depends on the interface between the channel material and the electrolyte solution. At a specific concentration of electrolyte solution, a double electric layer of a certain thickness forms at the sensing interface.^[^ ^75^, ^86^, ^87^ ^]^ Simultaneously, charged biomolecules in the solution will be coated with counter‐ions. This phenomenon, known as the Debye shielding, significantly affects Xenes FET biosensors by reducing the effective charge due to counter‐ion adsorption.^[^ ^28^, ^79^ ^]^ To quantify the impact of the Debye shielding effect on the effective charge of charged molecules, the concept of the Debye length is directly introduced. The numerical value of the Debye length reflects the effective distance over which charged molecules interact with the sensing interface.^[^ ^86^, ^88^ ^]^ The Debye length can be calculated using the formula provided in Equation (1).^[^ ^89^ ^]^

(1) $$\lambda_{D}=\sqrt{\frac{\varepsilon_{0}\varepsilon_{r}K_{B}T}{2N_{A}e^{2}I}}\;$$

where ε_0_ is the permittivity of free space; ε_*r*_ is the dielectric constant; *K_B_* is Boltzmann's constant; *T* is the temperature; *N_A_* is Avogadro's number; *e* is the elementary charge; and *I* is the ionic strength of the solution.

When the distance between the charged molecule and the sensing interface exceeds the Debye length, the Debye shielding effect causes a minimal change in channel conductivity due to the molecule, significantly reducing the biosensor's sensitivity. Conversely, when the distance between the charged molecule and the sensing interface is less than or equal to the Debye length, the charged molecule can effectively modulate the channel conductivity through its gating effect.^[^ ^80^ ^]^

### 2.4 Performance Indicators of Xenes FET Biosensors

The performance indicators including selectivity, sensitivity, stability, LOD, range of detection, and response time are important factors to take into account when assessing the performance of biosensors.^[^ ^28^, ^67^, ^90^, ^91^, ^92^ ^]^

#### 2.4.1 Selectivity

Non‐specific proteins,^[^ ^59^ ^]^ single‐base mismatched DNA,^[^ ^93^ ^]^ ions,^[^ ^70^ ^]^ and other chemicals or biomolecules^[^ ^83^, ^94^ ^]^ will typically be present in the detecting environment during the actual biomolecular detection process, in addition to the object that needs to be detected. Thus, one of the most crucial performance metrics for biosensors is their selectivity. The selectivity of biosensors is typically achieved through the specific binding of the target analyte to the bioreceptor present at the sensing interface, while concomitantly minimizing non‐specific interactions with other substances.^[^ ^91^ ^]^ There are numerous strategies to enhance the selectivity of biosensors, with the most prevalent approach being the utilization of an appropriate biological receptor to augment the specific binding interaction between the analyte and the receptor.^[^ ^95^ ^]^

#### 2.4.2 Sensitivity

Sensitivity is a parameter that reflects the biosensor's ability to detect and respond to small changes in the concentration of the substance to be detected. It is the change in amplitude of the sensor response signal relative to the concentration of the object to be detected.^[^ ^67^ ^]^ The sensitivity of the sensor is dependent on the Xenes present in the channel. The sensitivity of the biosensor can be visually reflected by the slope of the response signal and the concentration curve of the object to be detected.^[^ ^71^, ^83^ ^]^ The widespread consensus is that the sensor's sensitivity increases with the slope of the curve. When a functional relationship (*I_d_* =  *f*(*C*)) exists between the analyte concentration and the drain current, the specific value of sensitivity can be determined using the following formula^[^ ^96^ ^]^:

(2) $$\mathrm{Sensitivity}\;=\frac{I_{d^{\prime }}-I_{d}}{C^{\prime }-0}\;$$

where *C*′ is the concentration of the analyte, *I*_*d*′_corresponds to the drain current at *C*′, *I_d_* corresponds to the initial drain current.

#### 2.4.3 Response

To eliminate discrepancies between individual devices, the response signal is typically determined by the relative change in the test signal. Test signals such as drain current, Dirac voltage, and resistance are frequently utilized to calculate the sensor's response signal. The sensor's response can be calculated using the following formula:

(3) $$\mathrm{Response}\;=\frac{R_{i}-R_{0}}{R_{0}}\times 100\%$$

where *R*_0_ is the initial signal of the sensor and *R_i_* is the signal after the test.

#### 2.4.4 Response Time

Response time is an essential indicator for evaluating biosensors, defined as the duration needed for the electrical signal to achieve 95% of its equilibrium value after the introduction of the target analyte.^[^ ^92^ ^]^ A shorter response time is preferable for effective sensing. When sufficiently brief, the biosensor can facilitate real‐time detection of the targeted analyte.^[^ ^70^ ^]^

#### 2.4.5 Stability

An index that biosensors need to take into account is stability. Time and the detection environment are the two primary elements influencing the sensor's stability.^[^ ^90^ ^]^ To assess the sensor's stability, we present a method for calculating it. The subsequent formula details the computation of temporal stability.

(4) $$\mathrm{stabilit}y_{\mathrm{time}}=\frac{\mathrm{respons}e_{\mathrm{time}}-\mathrm{respons}e_{0}}{\mathrm{respons}e_{0}}\times 100\%$$

where response_0_ is the initial response signal of the sensor, and response_time_ is the response signal measured after a period of time.

The instability of black phosphorus and other Xenes in the presence of air and moisture constitutes a critical factor affecting device stability, necessitating protective encapsulation and passivation strategies.^[^ ^97^ ^]^ One encapsulation approach involves growing dense dielectric layers (e.g., HfO_2_ and Al_2_O_3_) via atomic layer deposition, which effectively isolates the materials from water/oxygen corrosion while suppressing spontaneous surface oxidation.^[^ ^98^ ^]^ The introduction of passivation layers (e.g., h‐BN) reduces the interfacial defect density between Xenes and the external environment,^[^ ^99^ ^]^ thereby minimizing carrier scattering and electrical signal drift.^[^ ^100^ ^]^ Additionally, surface modification with chemical compounds serves as an effective passivation method.^[^ ^44^ ^]^ For instance, constructing P─C covalent bonds through aryldiazonium salt‐mediated functionalization reactions significantly enhances the oxidation resistance of black phosphorus.^[^ ^101^ ^]^ These encapsulation and surface passivation techniques provide long‐term stability for Xenes, thus advancing their sensing applications in FET devices.

#### 2.4.6 LOD

The LOD refers to the lowest amount or concentration level at which the sensor can accurately measure the target analyte.^[^ ^28^ ^]^ The concept of noise level must be established before defining the LOD. In the context of Xenes FET biosensors, the noise level is characterized as thrice the signal elicited by the biosensor during the analysis of a blank sample. The LOD is identified as the concentration of the analyte that corresponds to the point where the noise level curve intersects the linear standard curve.^[^ ^71^ ^]^

#### 2.4.7 Range of Detection

Range of detection refers to the interval over which the detection signal maintains a linear correlation with the concentration of the target substance. It is important to note that the lower boundary of this range defines the detection limit, whereas the upper boundary corresponds to the concentration of the analyte that matches the apex of the linear curve.^[^ ^28^ ^]^

### 2.5 Potential Relationship Between FET Metrics and Biosensing Performance

The biosensing efficacy of Xenes‐based FETs is fundamentally governed by the synergistic optimization of intrinsic material properties, device‐level electrical parameters, and biointerface dynamics. First, the carrier mobility of Xenes directly dictates sensitivity by amplifying surface potential modulation induced by biomolecular adsorption, as quantified by the Nernst‐Poisson relationship.

High µ reduces threshold voltage noise floor to achieve sub‐femtomolar LOD. Second, gate‐tunable transconductance enables dynamic control over signal‐to‐noise ratios. Third, Xenes’ surface‐to‐volume ratio and defect‐engineered active sites synergistically influence response kinetics. Furthermore, the measurement reproducibility across repeated cycles can be ensured by minimizing the interface trap state in Xenes. These parametric interdependencies highlight the necessity of co‐engineering Xenes’ quantum confinement effects, dielectric interfaces, and biorecognition layers to unlock real‐time, multiplexed biodetection in complex matrices.

## 3 Structure, Preparation, Properties, and Theory of Xenes

The 3D material typically serves as the channel material for conventional FET sensors.^[^ ^10^, ^102^, ^103^, ^104^ ^]^ However, due to the inherent limitations of the 3D structure, channel modulation is restricted. Consequently, environmental changes primarily affect the electrical characteristics of the material's upper surface, resulting in relatively low signal conversion efficiency.^[^ ^30^, ^31^ ^]^ FET sensor research has been concentrating on substituting low‐dimensional materials for 3D channel materials to address these issues. Xenes offer several significant advantages over traditional 3D materials, including a large specific surface area,^[^ ^66^ ^]^ low sheet resistance,^[^ ^105^, ^106^, ^107^ ^]^ and excellent carrier mobility.^[^ ^108^, ^109^, ^110^ ^]^ These properties make Xenes particularly well‐suited for enhancing the performance of FET sensors. The development of FET sensors based on Xenes has advanced significantly in the last few years.^[^ ^80^, ^111^ ^]^

**Figure 6:**
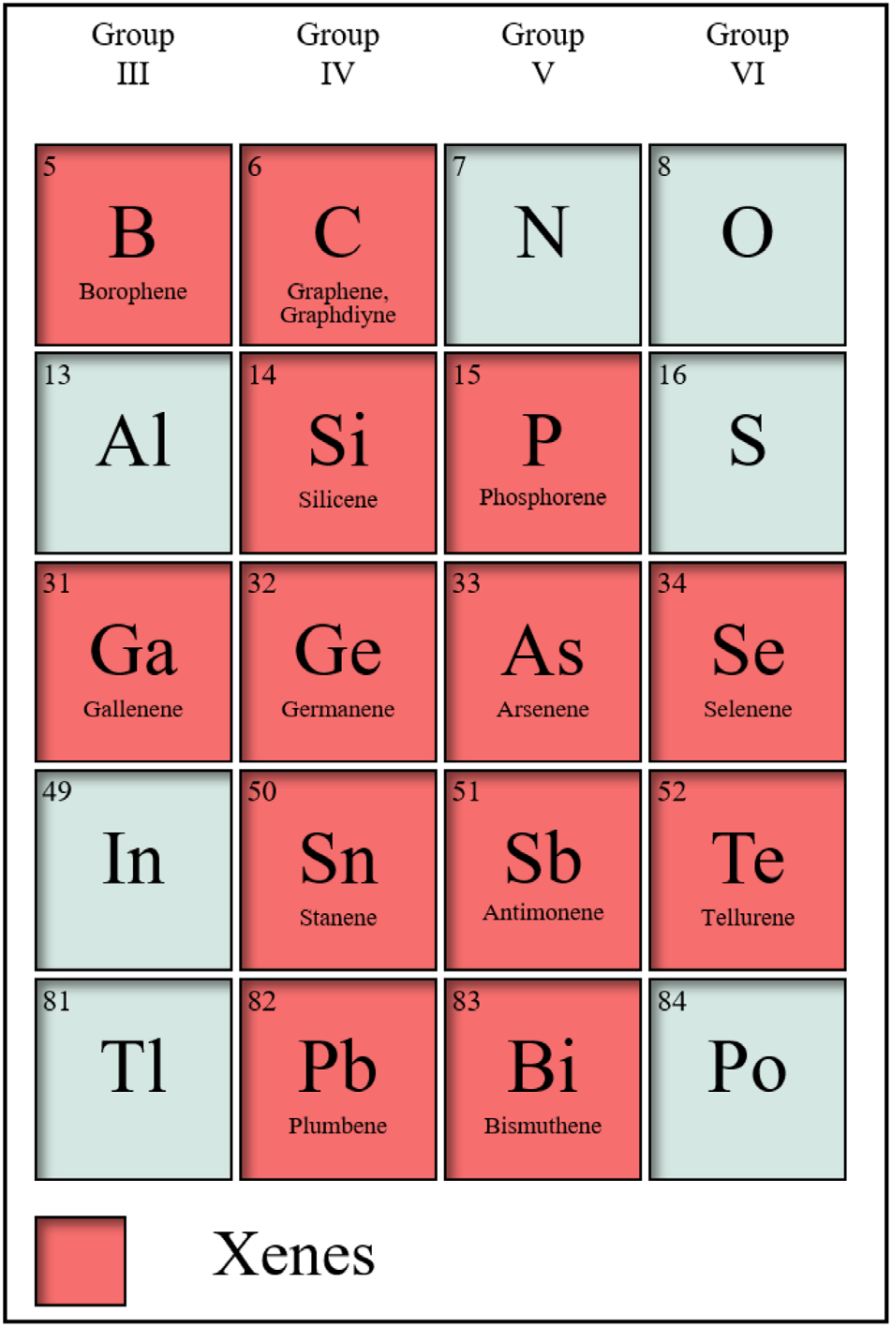
The types of Xenes and the position of Xenes in the periodic table.

For the convenience of discussion, we have classified Xenes. Based on their positions in the periodic table, they can be categorized into: Group III (including borophene and gallenene), Group IV (encompassing graphene, graphdiyne, silicene, germanene, stanene, and plumbene), Group V (comprising phosphorene, arsenene, antimonene, and bismuthene), and Group VI (consisting of selenene and tellurene). **Figure** **6** displays all the Xenes that have been successfully synthesized. **Table** **1** provides a summary of the pertinent parameters associated with Xenes. Nonetheless, it should be noted that not all Xenes are currently applicable for FET biosensors. Consequently, this section focuses primarily on those Xenes that have been employed in the construction of FET, with an in‐depth examination of their structural features, synthesis procedures, and crucial properties.

**Table 1:** Summary of the basic parameters of various Xenes. where *a* and *b* are lattice constants, *h* is the buckling height, and *α* is the Angle between neighboring bonds.

Xenes	Bandgap [eV]	Carrier mobility [cm^2^ V^−1^ s^−1^]	*a* [Å]	*b* [Å]	*h* [Å]	*α* [°]	Young's modulus	Refs.
Borophene	Metal	X: 14.8 × 10^5^, Y: 28.4 × 10^5^	1.614	2.866	0.911	94.0	E_a_ = 389 N m^−1^, E_b_ = 166 N m^−1^	[112, 113]
Gallenene (a_100_)	Metal	–	2.72	2.54	0	α_1_ = 139.54, α_2_ = 105.43, α_3_ = 115.03	–	[114]
Gallenene (b_010_)	Metal	–	2.77	2.72	1.32	α_1_ = 111.64, α_2_ = 115.03	–	[114]
Graphene	Zero bandgap	≈3750	2.46	2.46	–	120	1 TPa	[115, 116]
Graphdiyne	0.46	10^4^–10^5^	16.42	9.48	–	–	–	[117]
Silicene	Zero bandgap	≈100	3.868	2.233	0.44	116.3	152.93 GPa	[118, 119]
Germanene	0.79	≈10^4^	3.97	2.344	0.65	112.3	E_a_ = 90.9 N m^−1^, E_b_ = 92.7 N m^−1^	[44, 120]
Stanene	0.15	(3‐4) × 10^6^	4.62	2.82	0.92	110.0	46.3 ± 0.2 GPa	[41, 121]
Plumbene	1.34	–	4.9257	2.9906	0.9256	–	–	[122]
Phosphorene	0.3‐2.0	≈10^3^	4.52	3.31	–	102	E_a_ = 167.88 N m^−1^, E_b_ = 39.39 N m^−1^	[44, 76]
Arsenene	1.2‐1.4	≈10^3^	2.45	2.45	0.64	92.54	21.5 N m^−1^	[43, 123]
Antimonene	0‐2.28	≈10^3^	–	–	2.2	–	–	[124]
α‐Bismuthene	0.36	≈10^3^	–	–	–	–	–	[125]
β‐Bismuthene	0.99	≈10^3^	–	–	–	–	–	[125]
Selenene	0.1	–	3.65	–	0.77	–	–	[126]
α‐Tellurene	0.75	≈10^3^	4.15	4.15	3.67	–	–	[127]
β‐Tellurene	1.47	≈10^3^	4.17	5.49	2.16	–	–	[127]

### 3.1 Structure of Xenes

In this section, the crystal structure and basic properties of various Xenes are briefly described.

#### 3.1.1 Graphene

**Figure 7:**
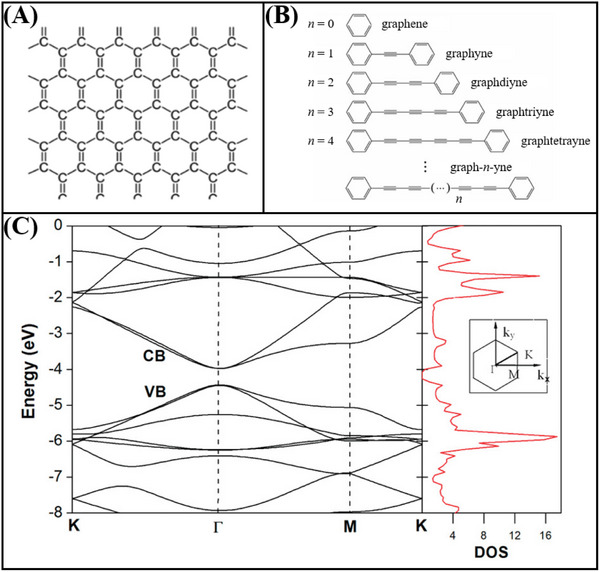
A) The lattice structure of graphene.^[^ ^130^ ^]^ Copyright 2020, Elsevier. B) Schematic structures of “graphynes”.^[^ ^138^ ^]^ Copyright 2012, Royal Society of Chemistry. C) Typical band structure and state density of graphdiyne.^[^ ^117^ ^]^ Copyright 2011, American Chemical Society.

Graphene, the first 2D material to be isolated, is characterized by a solitary layer of carbon atoms bonded via sp^2^ hybridization,^[^ ^128^ ^]^ which results in a distinctive hexagonal lattice structure.^[^ ^129^ ^]^ This configuration is elucidated in **Figure** **7**A.^[^ ^130^ ^]^ As a typical 2D material, graphene has many excellent properties. First of all, it boasts remarkable mechanical attributes; the failure strength of a monolayer of graphene is recorded at 42 N m^−1^, with its Young's modulus potentially reaching up to 1.0 TPa.^[^ ^131^ ^]^ Second, graphene exhibits extraordinary electrical conductivity. The thermal conductivity of a monolayer of suspended graphene has been measured at 5,000 W mK^−1^,^[^ ^132^ ^]^ a testament to its exceptional thermal conductivity, which can be attributed to its repetitive structure and the relatively low mass of carbon atoms.^[^ ^133^ ^]^ Third, graphene's optical properties are also noteworthy, as its unique electronic structure enables an absorption rate of incident white light at ≈2.3%.^[^ ^134^ ^]^ Finally, graphene's conduction band base and valence band top degenerate at the Dirac point, thus becoming a zero‐bandgap semiconductor.^[^ ^130^ ^]^ This distinctive electronic structure endows graphene with exceptionally high conductivity. However, despite its superior performance, graphene's zero‐bandgap and chemical inertness limit its applications in FET sensing and optical sensing.^[^ ^42^, ^135^ ^]^ Additionally, graphene faces several challenges, particularly in large‐scale production, which can affect the consistency and quality of the material. Integrating graphene into commercial products remains a complex task, as researchers work to address these issues through various synthesis and processing techniques.

#### 3.1.2 Graphdiyne

Graphynes constitute a novel group of 2D carbon materials made up of carbon atoms in both sp‐ and sp^2^‐ hybridization states.^[^ ^136^ ^]^ Indeed, “graphynes” exhibit a variety of structural configurations. They are differentiated into graphyne, graphdiyne, and graph‐*n*‐yne based on the quantity of “─C≡C─” bonds that link adjacent sp^2^‐ hybridized carbon atoms (Figure 7B).^[^ ^137^, ^138^ ^]^ Unlike graphene, a zero‐bandgap semimetal, the electronic structure of graphdiyne presents a tunable bandgap due to the different bonding configurations of sp‐ and sp^2^‐ hybridized carbon atoms, with graphdiyne being considered an intrinsic direct‐bandgap semiconductor. The typical band structure and density of states of graphdiyne are illustrated in Figure 7C. The bandgap at the Γ‐point is 0.46 eV.^[^ ^117^ ^]^ Zheng et al.^[^ ^139^ ^]^ conducted a systematic investigation into the structural and electronic properties of bilayer and trilayer graphdiyne using first‐principles calculations based on DFT. Their findings revealed that the most stable configuration of bilayer graphdiyne has a bandgap of 0.35 eV, while the trilayer graphdiyne exhibits a bandgap of 0.33 eV, both of which are smaller than the intrinsic bandgap of monolayer graphdiyne. The superior electronic properties, tunable bandgap, excellent surface activity, and the flexibility in fabrication and manipulation of graphdiyne make it a promising material for applications in FET sensor technology.

#### 3.1.3 Silicene

**Figure 8:**
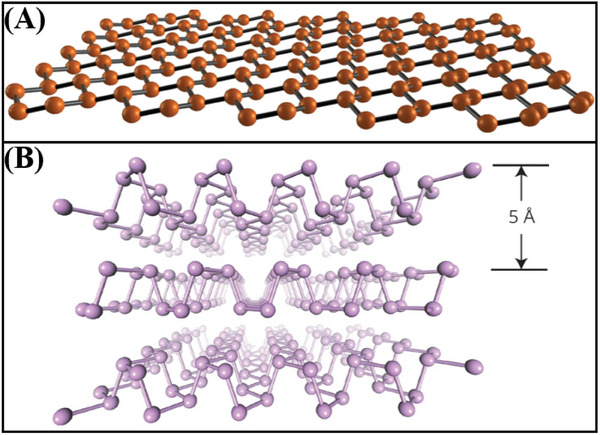
A) Curved cellular lattice structure of silicene.^[^ ^141^ ^]^ Copyright 2015, Springer Nature. B) Schematic diagram of 2D BP structure with folded structure.^[^ ^76^ ^]^ Copyright 2014, Springer Nature.

The inception of silicene research dates back to 2007, when Guzman et al.^[^ ^140^ ^]^ formally defined silicene as a 2D monolayer of silicon atoms arranged in a sp^2^ hybridized honeycomb lattice. They devised a unified tight‐binding models to characterize the electronic properties of this novel material. Computational analyses based on this model have classified silicene as a zero‐bandgap semiconductor. Silicene exhibits a honeycomb crystal structure, as shown in **Figure** **8**A.^[^ ^141^ ^]^ Feng et al.^[^ ^142^ ^]^ were able to grow large‐area, continuous silicene on Ag(111) surfaces and directly observed its unique honeycomb lattice structure using scanning tunneling microscopy. Compared to graphene, silicene's significant advantage lies in its stronger spin‐orbit coupling, which results in a substantial bandgap at the Dirac point, potentially leading to an observable quantum spin Hall effect.^[^ ^41^ ^]^ Additionally, the bandgap of silicene can be significantly opened through chemical modification methods, which is crucial for applications in devices such as FETs. With a Dirac electronic structure similar to that of graphene, silicene holds potential for applications in electronic devices, optoelectronics, chemical and biological sensors, and energy storage. Moreover, the tunability of silicene offers further possibilities for achieving high‐performance semiconductor devices.^[^ ^119^ ^]^

#### 3.1.4 Phosphorene

Phosphorus can exist in several amorphous forms, including white phosphorus, red phosphorus, blue phosphorus, violet phosphorus, and black phosphorus (BP).^[^ ^45^ ^]^ BP is the most thermodynamically stable allotrope form under environmental conditions.^[^ ^123^ ^]^ As such, the crystal structure and electronic structure of black phosphorene are primarily discussed here. Figure 8B depicts the crystal structure of BP.^[^ ^76^ ^]^ The unit cell of BP has eight atoms with a density of 2.69 g cm^3^. BP features a single crystal cell consisting of two layers, wherein each phosphorus atom is bonded to three neighboring phosphorus atoms. The bond length between these atoms is 2.18 Å, the bond angles are 103°, 103°, and 99°, and the average bond angle is 102°.^[^ ^143^ ^]^ BP possesses a direct bandgap, the magnitude of which is dependent on the number of layers present. This bandgap can be adjusted from 0.3 to 2 eV by changing the thickness of the BP.^[^ ^42^ ^]^ In addition, the carrier mobility of 2D BP can reach 1000 cm^2^V^−1^s^−1^,^[^ ^76^ ^]^ with excellent mechanical properties, and anisotropic optical properties.^[^ ^45^ ^]^ Consequently, BP possesses a broad range of applications in photoelectric devices.^[^ ^144^ ^]^

#### 3.1.5 Tellurene

The 2D monolayer structure of tellurene is predicted using particle swarm optimization and first‐principles DFT. Tellurene exhibits a stable 1T‐MoS_2_‐like (α‐Te) structure, as well as metastable tetragonal (β‐Te) and 2H‐MoS_2_‐like (γ‐Te) structures.^[^ ^127^ ^]^ α‐Te and β‐Te are semiconductors characterized by bandgaps of 0.75 and 1.47 eV, respectively. They exhibit carrier mobilities up to 10^3^ cm^2^V^−1^s^−1^. Furthermore, these materials possess a light absorption rate exceeding 10^5^ cm^−1^. It is also noteworthy that β‐Te displays anisotropy in its electronic and optical properties.^[^ ^145^ ^]^ Tellurene possesses a suite of advantageous properties, including high carrier mobility, exceptional light absorption capabilities, superior thermoelectric characteristics, and stable performance at ambient temperatures, rendering it a promising material for applications in nanodevices.^[^ ^146^, ^147^ ^]^

### 3.2 Preparation of Xenes

As research has advanced, Xenes preparation technology has steadily grown and improved. It can be categorized into two approaches: “bottom‐up” and “top‐down”.^[^ ^45^ ^]^

#### 3.2.1 Bottom‐up Methods

The bottom‐up approach to material preparation entails the fabrication of materials from the molecular or atomic level, typically incorporating chemical reactions and self‐assembly processes.^[^ ^148^ ^]^ The bottom‐up method mainly includes chemical vapor deposition (CVD) and molecular beam epitaxy (MBE).

**Figure 9:**
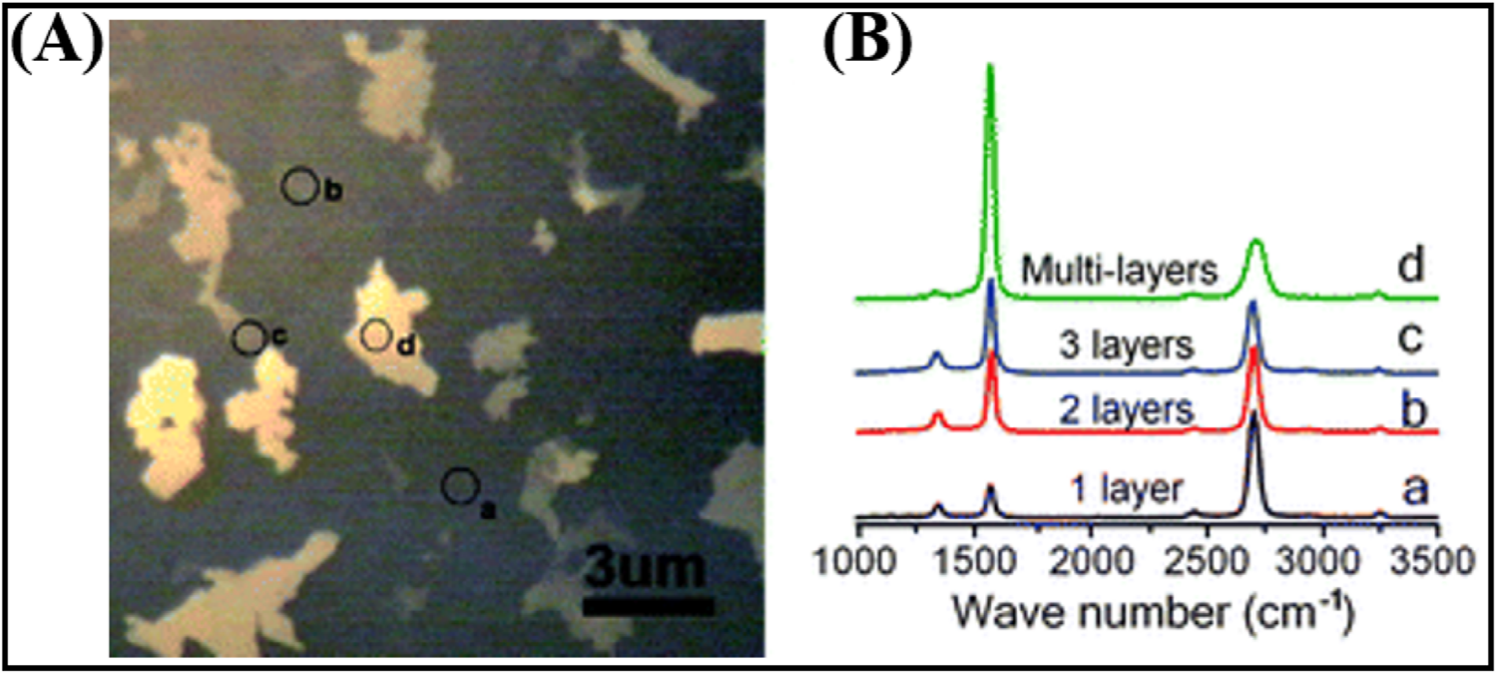
(A) Optical images of graphene films prepared by CVD. (B) Raman spectra of different thicknesses of graphene.^[^ ^151^ ^]^ Copyright 2010, Royal Society of Chemistry.

The process known as CVD uses gaseous precursors to react chemically on a substrate to create the desired thin‐film material. In CVD, the thickness and mass of the film can be precisely controlled at the atomic level by adjusting reaction conditions such as temperature, pressure, and gas flow rate.^[^ ^149^ ^]^ The technology of CVD is employed in the synthesis of Xenes due to its versatile deposition capabilities, precise control over composition, enhanced crystalline properties, and superior coverage ability.^[^ ^150^ ^]^ Huang et al.^[^ ^151^ ^]^ successfully cultivated large‐scale graphene films, characterized by both single and few‐layer domains, on nickel substrates utilizing the CVD technique at a temperature of 1000 °C. The carbon source for this process was a mixture composed of methane and hydrogen. The formation of graphene films was confirmed through optical microscopy (**Figure** **9**A) and Raman spectroscopy (Figure 9B) analyses, thereby corroborating the efficacy of the growth process. The thickness of CVD‐prepared graphene can be approximately determined by the characteristic intensity ratio of the 2D Raman peak at ≈2700 cm^−1^ to the G peak at ≈1582 cm^−1^. Specifically, region a corresponds to monolayer graphene with a characteristic peak intensity ratio of ∼3.4, while region d corresponds to multilayer graphene with a characteristic peak intensity ratio of ≈0.25. CVD is not solely utilized for the synthesis of graphene; it also serves as a method for producing other single‐element 2D materials. Smith et al.^[^ ^152^ ^]^ utilized red phosphorus as the starting material to synthesize a few layers of substrate black phosphorus (SBP) on a silicon substrate via CVD. AFM characterization revealed that the resulting BP sample exhibited distinct layering. The thickness of a single layer was determined to be 0.85 nm, while that of a quadruple layer was 3.4 nm, with an area of 0.35 µm^2^ for the four‐layered section. Raman spectroscopy was employed to verify the quality of the SBP, revealing characteristic peaks at 365, 442, and 470 cm^−1^, which indicate the successful fabrication of BP films. Tan et al.^[^ ^153^ ^]^ introduced atomically flat hexagonal boron nitride nanosheets as growth substrates into the VCD system, achieving the synthesis of high‐quality single‐crystalline tellurium nanobelts. The tellurium‐based field‐effect transistors exhibited an ultrahigh hole mobility of 1370 cm^2^V^−1^s^−1^ at room temperature. Although CVD technology demonstrates promising development prospects, its dependence on lattice‐matched substrates currently enables the preparation of only a limited number of Xenes via this method.^[^ ^44^, ^154^ ^]^ Therefore, further exploration of CVD is required.

MBE is an advanced technique for film deposition, executed in an ultra‐high vacuum.^[^ ^155^ ^]^ The fundamental principle involves the directed impingement of one or multiple thermalized molecular (or atomic) beams onto a crystalline substrate, resulting in physical deposition on the substrate's surface and subsequent film growth.^[^ ^156^ ^]^ The ultra‐high vacuum environment ensures that the molecular beam encounters minimal collisions with residual gases before substrate contact, thereby maintaining the high purity and precise control characteristic of the MBE process.^[^ ^41^ ^]^ MBE is the main method of borophene growth.^[^ ^157^ ^]^ Feng et al.^[^ ^158^ ^]^ employed molecular beam epitaxy to synthesize borophene by depositing it on the surface of an Ag (111) single crystal via direct evaporation from a pristine boron source. Experimental findings, coupled with first‐principles calculations, have substantiated the formation of two distinct borophene structures on the Ag (111) surface: the β_12_ sheet and the χ_3_ sheet. Notably, the borophene synthesized via molecular beam epitaxy exhibited remarkable resistance to oxidation and engaged in minimal interaction with the Ag (111) substrate. Li et al.^[^ ^141^ ^]^ grew silicene on Ag (111) films by MBE method, and constructed a silicene FET based on the prepared silicene for the first time. The silicene FET exhibits ohmic contact under ambient conditions, and its transfer characteristic curves align with the commonly employed bipolar diffusion transport model. In addition to borophene and silicene, various Xenes materials can be synthesized using MBE, including graphene,^[^ ^155^ ^]^ stanene,^[^ ^159^ ^]^ bismuthene,^[^ ^160^ ^]^ and antimonene.^[^ ^124^ ^]^

#### 3.2.2 Top‐down Methods

The top‐down method for material preparation typically involves the reduction of a bulk crystal to the desired dimensions through external means. It should be mentioned that only Xenes with layered composite blocky crystals are good candidates for the top‐down method.^[^ ^148^ ^]^ The top‐down method is represented by mechanical exfoliation and liquid phase exfoliation.

Mechanical exfoliation is a foundational technique for the preparation of 2D materials, which relies on mechanical forces to separate layered materials.^[^ ^148^ ^]^ Owing to the absence of chemical reagents and reactions in the mechanical exfoliation process, the resulting material possesses high purity with minimal impurities.^[^ ^41^, ^161^ ^]^ Novoselov et al.^[^ ^162^ ^]^ pioneered the preparation of graphene through mechanical exfoliation. Utilizing adhesive tape, they disrupted the van der Waals forces binding the layers of highly oriented pyrolytic graphite, culminating in the isolation of a monolayer graphene with a thickness of ≈0.8 nm. Sun et al.^[^ ^163^ ^]^ introduced a method for the preparation of large‐area 2D crystals, utilizing an Au‐assisted mechanical exfoliation method. In this process, the tape, bearing the massive crystals, is brought into contact with a substrate pre‐coated with gold. A specific amount of pressure is then applied to ensure optimal contact. After an allotted time, the tape is carefully peeled away, resulting in one or several large single‐layer flakes adhered to the gold surface. The findings indicate that the BP monolayer, synthesized using this methodology, exhibits superior quality with an approximate thickness of 0.6 nm. Xenes such as arsenene,^[^ ^164^ ^]^ bismuthine,^[^ ^165^ ^]^ and antimonene^[^ ^166^ ^]^ can also be prepared by mechanical stripping. In general, mechanical exfoliation serves as a fundamental technique for the preparation of 2D materials and is extensively employed in laboratory settings due to its simplicity. Nevertheless, for industrial‐scale production or applications necessitating high precision, more sophisticated technologies might be necessary to fulfill the requisite standards.

**Figure 10:**
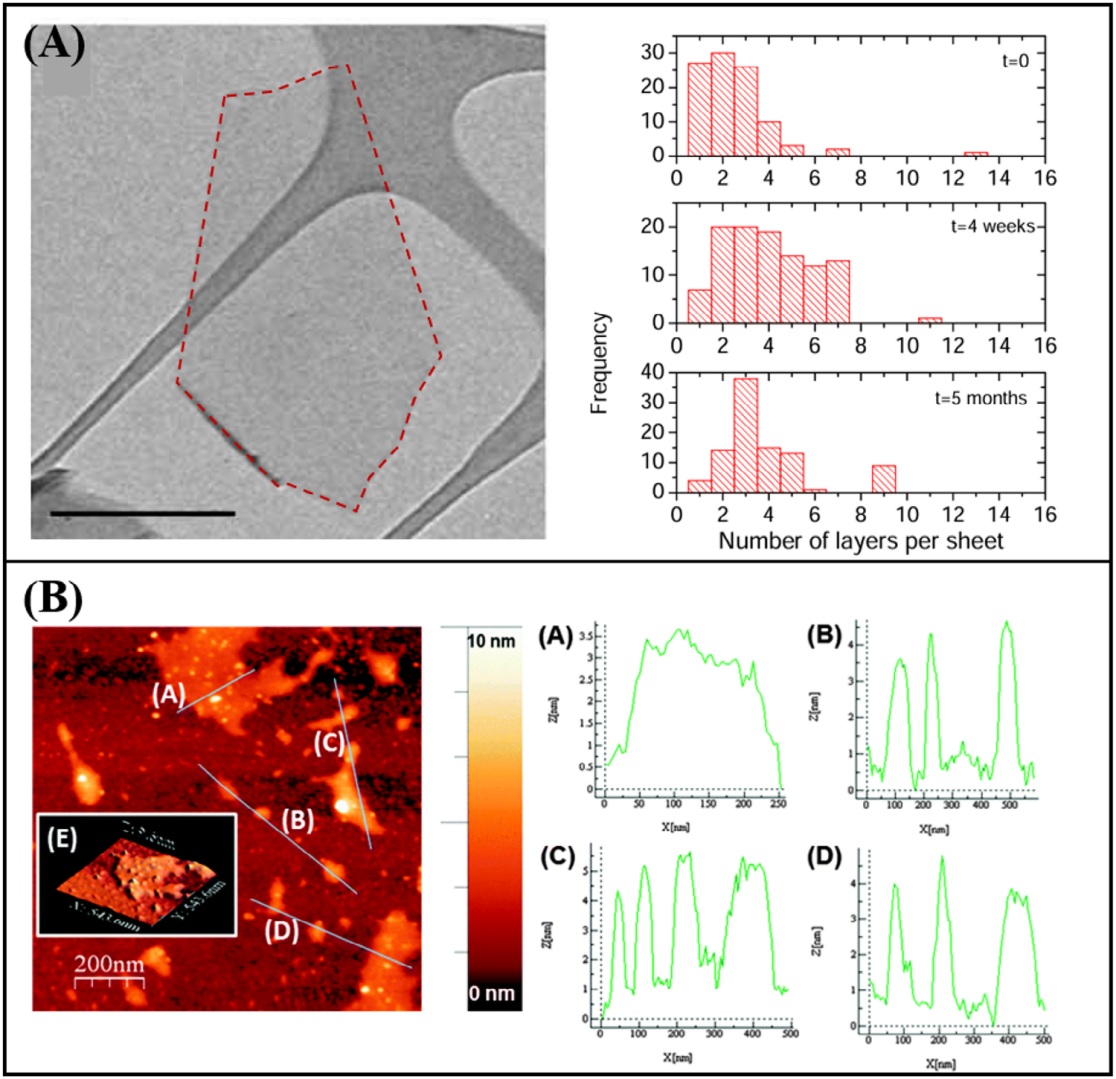
A) TEM image of monolayer graphene prepared by liquid phase stripping method.^[^ ^173^ ^]^ Copyright 2009, WILEY‐VCH. B) AFM image of BP prepared by liquid phase stripping method.^[^ ^168^ ^]^ Copyright 2014, Royal Society of Chemistry.

Liquid phase exfoliation is another popular technique. When Xenes are dispersed in a liquid phase, they typically expand between their body layers, causing the intermolecular van der Waals force to decrease. Once this force is reduced, ultrasonic vibrations provide energy to further disperse the layers, ultimately accomplishing the goal of stripping the material.^[^ ^161^ ^]^ Different liquid phase solvents exhibit varying degrees of stripping efficiency; therefore, the solvent must be selected based on the target material.^[^ ^148^ ^]^ Currently, N‐methylpyrrolidone (NMP) is the predominantly utilized solvent for the liquid phase stripping of graphene and BP. Hernandez et al.^[^ ^167^ ^]^ dispersed graphite powder in NMP, subjecting it to ultrasonic treatment followed by centrifugation. Monolayer graphene was successfully obtained without the introduction of oxides or structural defects during the liquid‐phase exfoliation process (**Figure** **10**A). Extensive data analysis reveals that the number fraction of monolayer graphene in NMP dispersions is 28%. Moreover, the monolayer graphene prepared by this method demonstrates excellent stability, with the monolayers remaining dispersed and not aggregating over an extended period (Figure 10B). Using liquid phase exfoliation of BP in NMP, Brent et al.^[^ ^168^ ^]^ prepared samples containing three to five layers of phosphorene with significant lateral dimensions, as well as one to three layers of phosphorene. The BP obtained through this method was characterized using AFM. The thickness of the larger nanosheets was ≈3.5–5 nm, while the single‐layer phosphorene exhibited a thickness of ≈0.9 nm. NMP's high boiling point and some toxicity, however, prevent it from being used much longer. Tyurnina et al.^[^ ^169^ ^]^ have suggested peeling graphene with a solvent mixture consisting of water, ethanol, and sodium cholate to address the solvent issue. With a very stable solution that can hold 78% of the flakes in the suspension after three months, this approach can produce graphene with a yield as high as 9%. Borophene,^[^ ^170^ ^]^ antimonene,^[^ ^171^ ^]^ and bismuthene^[^ ^172^ ^]^ can also be synthesized using liquid phase exfoliation.

In summary, the preparation methods for Xenes encompass a precisely controlled bottom‐up approach and a comparatively straightforward top‐down approach. Each method possesses its own set of merits and drawbacks, allowing researchers to select the most suitable preparation technique by the intended application and performance criteria.

### 3.3 Properties of Xenes

Numerous Xenes have been the subject of in‐depth research recently, leading to the prediction or confirmation of their varied features. The general physical, chemical, electrical, and optical properties of Xenes are outlined in this section.

#### 3.3.1 Physical Properties

Xenes has numerous special physical features not found in other non‐2D materials since it is composed of a single layer of atoms.^[^ ^174^ ^]^ The first and most noticeable property of Xenes is its extraordinarily enormous specific surface area, which is mostly attributable to its incredibly thin 2D structure. Because of its high loading capacity and large specific surface area, Xenes is more suited for the modification of biomolecules on its surface, which is advantageous for the Xenes FET biosensor.^[^ ^45^ ^]^ Moreover, the diverse bulk structures of Xenes present distinct variations, thereby offering a wider array of synthetic approaches for the preparation of Xenes.^[^ ^61^ ^]^

#### 3.3.2 Chemical Properties

The material's surface functionalization and the degradability of the biological system are the primary indicators of Xenes' chemical properties.^[^ ^10^, ^28^, ^51^ ^]^ First, Xenes' surface functionalization enables the attachment of biomarkers, which has numerous uses in the biosensor industry. As an exemplary interfacial coupling agent for probe linkers, one end of 1‐pyrene butyrate n‐hydroxysuccinimide can be changed on graphene by π‐π stacking interactions.^[^ ^70^, ^82^ ^]^ With further chemical functionalization, the analytical range and sensitivity of Xenes FET biosensors can be tuned and versatile.^[^ ^46^ ^]^ Moreover, the biodegradability of Xenes in biological systems is a crucial characteristic of their application in biosensors. Some Xenes materials exhibit reduced stability under environmental conditions, which could be advantageous for biosensor applications due to their facile degradation and metabolism within biological systems.^[^ ^45^ ^]^ Some of xenon's poor stability issues can be addressed through passivation or the incorporation of covalent functional groups.^[^ ^61^ ^]^

#### 3.3.3 Electronic Properties

Xenes has been extensively researched for its electrical applications due to its multitude of compelling properties. First, the majority of Xenes exhibit high carrier mobility.^[^ ^41^ ^]^ For instance, BP has a carrier mobility of ≈10^3^ cm^2^V^−1^s^−1^,^[^ ^42^ ^]^ while tellurene can reach up to 10^5^ cm^2^V^−1^s^−1^.^[^ ^175^ ^]^ The higher mobility of carriers in Xenes materials facilitates their rapid movement and charge conduction, which is crucial for the design and performance of high‐speed electronic devices. Moreover, the electronic properties of the majority of Xenes can be modulated through external means (changing the material's thickness, applying an external electric field, tensile strain, etc.^[^ ^46^ ^]^). Both BP ^[^ ^42^ ^]^ and bismuthene^[^ ^176^ ^]^ possess tunable direct bandgaps, the magnitudes of which are contingent upon their respective thicknesses. The application of an external electric field serves as an efficient method for band‐gap modulation. Under the influence of this external electric field, the bandgap of germanene can be modulated linearly while preserving the integrity of the Dirac cone.^[^ ^177^ ^]^ Typically, arsenic and antimony exhibit semi‐metallic properties; however, they transform indirect semiconductors with respective bandgaps of 2.49 and 2.28 eV when reduced to a single atomic layer. The application of tensile strain enables arsenene and antimonene to transition from indirect to direct bandgap semiconductors.^[^ ^43^ ^]^ In summary, the electronic properties of Xenes are primarily characterized by their high electron mobility and adjustable electronic characteristics. The unique electronic characteristics of Xenes make them highly promising for significant applications in the field of biosensing.

#### 3.3.4 Optical Properties

Xenes possess unique optical properties, which significantly broaden their potential applications. The optical anisotropy observed in Xenes is a ubiquitous trait, intimately linked to the inherent structural anisotropy of these materials.^[^ ^46^ ^]^ For instance, the pronounced in‐plane optical anisotropy in borophene stems from its directionally dependent crystalline structure. This results in significant variations in the absorption coefficient and reflectance along different orientations.^[^ ^178^ ^]^ Tran et al.^[^ ^179^ ^]^ employed first‐principles calculations to forecast the markedly anisotropic optical behavior of BP. In their study, they noted that multilayer BP exhibits intense absorption for light polarized parallel to the armchair direction of its lattice, whereas it remains virtually transparent to light polarized along the zigzag direction. Furthermore, most Xenes exhibit a broad absorption spectrum. Antimonene demonstrates an extensive photoresponse spanning from the infrared region to a peak in the ultraviolet domain.^[^ ^180^ ^]^ Additionally, antimonene displays a pronounced photoluminescence peak within the visible spectrum. Similarly, arsenene possesses multiple robust absorption peaks across the ultraviolet and visible spectra.^[^ ^46^ ^]^ The nonlinear optical properties of Xenes are noteworthy.^[^ ^41^ ^]^ Under intense incident light, the photogenerated carrier concentration surpasses the intrinsic concentration. Consequently, these photogenerated carriers occupy an empty state, impeding the flow of carriers within the band and leading to diminished light absorption. Owing to this characteristic, materials like phosphorene^[^ ^181^ ^]^ and antimonene are frequently employed as saturable absorbers in mode‐locked lasers.

Taken together, the optical properties of Xenes not only offer novel possibilities for the design and fabrication of optoelectronic devices but also present fresh insights into the advancement of biomedical and biosensing technologies.

### 3.4 Theory of Xenes

Theoretical calculations play an indispensable role in understanding Xenes materials and their biosensing applications.^[^ ^182^ ^]^ Through computational methods such as DFT, researchers can reveal critical properties of Xenes at the atomic scale, including their electronic structures and regulatory mechanisms of band structures.^[^ ^48^ ^]^ These calculations not only explain experimentally observed electrical behaviors but also predict the influence of surface states and edge effects on charge distribution, providing theoretical guidance for designing high‐mobility devices.^[^ ^49^ ^]^ The significance of theoretical calculations further lies in their ability to overcome experimental limitations. Atomic‐level simulations address the shortcomings of traditional characterization techniques in dynamically monitoring interfacial reactions. High‐throughput theoretical calculations enable rapid screening of Xenes derivatives to predict key parameters such as bandgap and adsorption energy. Such a “computation‐before‐experiment” strategy significantly reduces experimental trial‐and‐error costs.

In biosensing applications, the synergistic interplay between theoretical models and experiments is particularly notable. For example, Song et al.^[^ ^183^ ^]^ demonstrated the high affinity between 11‐mercaptoundecanoic acid and material surfaces through DFT, offering a theoretical basis for sensor surface functionalization. Babolghani et al.^[^ ^184^ ^]^ simulated the detection of polycyclic aromatic hydrocarbons using FET biosensors via DFT calculations and elucidated the charge transfer processes. These cases highlight how theoretical calculations not only deepen the understanding of materials’ intrinsic properties but also bridge microscopic mechanisms with macroscopic performance, facilitating the transition of Xenes‐based sensors from empirical exploration to rational design.

## 4 Applications of Biosensors

Based on the aforementioned examination of Xenes' structure and characteristics, we have enumerated the primary factors contributing to its extensive potential for use in the biosensing industry as follows: 1) The sensor's performance can be improved by the ultra‐high specific surface area of Xenes, which can offer more active sites, increase load efficiency, and make chemical modification and molecular functionalization easier;^[^ ^79^, ^185^, ^186^ ^]^ 2) Compared with 2D materials with planar architectures, some pleated Xenes exhibit a higher specific surface area, thereby enabling shorter required sensing channel lengths or, equivalently, demonstrating a reduced biosensing channel length limit. The shortened channel length will diminish electron transfer time and accelerate biosensing speed;^[^ ^187^, ^188^, ^189^ ^]^ 3) The unique surface of Xenes with non‐bonding electrons allows its physical and chemical properties to be easily regulated and supports flexible electrochemical reactions with different chemical functional groups;^[^ ^190^, ^191^ ^]^ 4) The alloy strategy based on Xenes has the potential to reduce the LOD;^[^ ^44^ ^]^ 5) It has been demonstrated that Xenes, which possess exceptional mechanical strength and flexibility, can continue to function even when subjected to significant structural deformation. Moreover, such structural deformation can be utilized to extend the effective Debye length, thereby facilitating signal acquisition in biosensing;^[^ ^187^ ^]^ 6) Due to the relatively basic structure and atomic composition of Xenes, precise theoretical studies are easier to achieve, which allows the exploration of new mechanisms in related applications.^[^ ^192^, ^193^, ^194^ ^]^ In conclusion, Xenes are regarded as the star material with the potential of high sensitivity, high flexibility, and low detection limits in the field of future biosensors because of its distinct structural, electrical, and other properties. Furthermore, Xenes exhibit superior sensitivity and response speed compared to commercial materials such as single‐crystalline silicon, polycrystalline silicon, ZnO, IGZO, pentacene, and P3HT.^[^ ^195^ ^]^ In the field of flexible sensors, the stability of Xenes is anticipated to rival that of commercial organic semiconductors like pentacene and P3HT.^[^ ^196^ ^]^

In recent years, Xenes FET biosensors have been extensively utilized and developed. In this section, the target analytes are categorized into four groups: nucleic acids, proteins, small biomolecules, and cells, and the current advancements in Xenes FET biosensors are summarized.^[^ ^28^ ^]^

### 4.1 Nucleic Acid Sensors

Nucleic acids, such as DNA and RNA, serve as carriers of genetic information and are indispensable in biological systems, playing a pivotal role in various biological processes.^[^ ^10^, ^197^, ^198^ ^]^ In the context of diseases, nucleic acids act as biomarkers that emerge before antibodies, rendering nucleic acid testing superior for the early diagnosis of diseases.^[^ ^67^ ^]^

#### 4.1.1 Graphene

**Figure 11:**
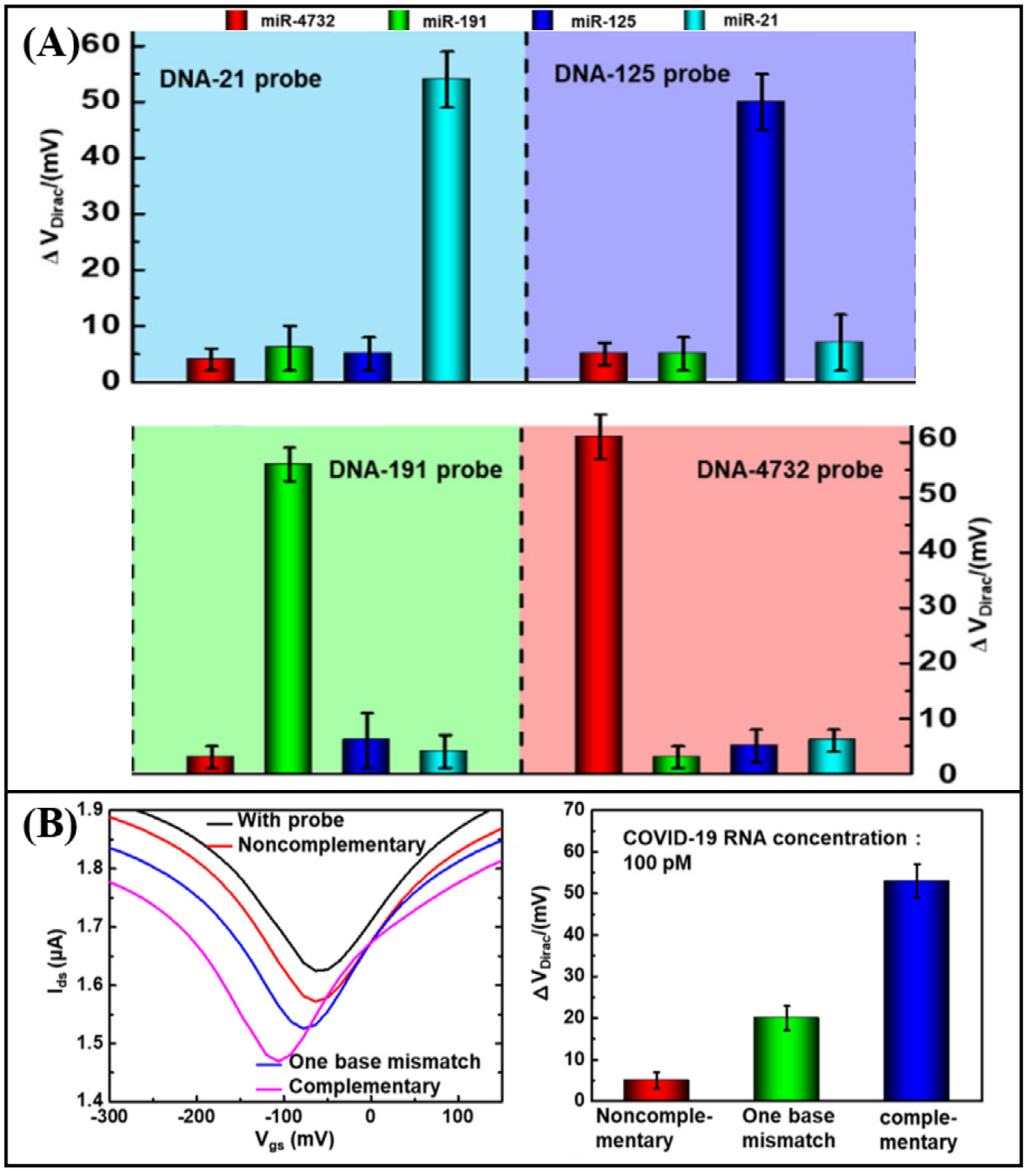
A) Graphene FET biosensor for miRNA detection. B) Graphene FET biosensor for the detection of SARS‐CoV‐2 RNA.^[^ ^81^ ^]^ Copyright 2022, American Chemical Society.

**Figure 12:**
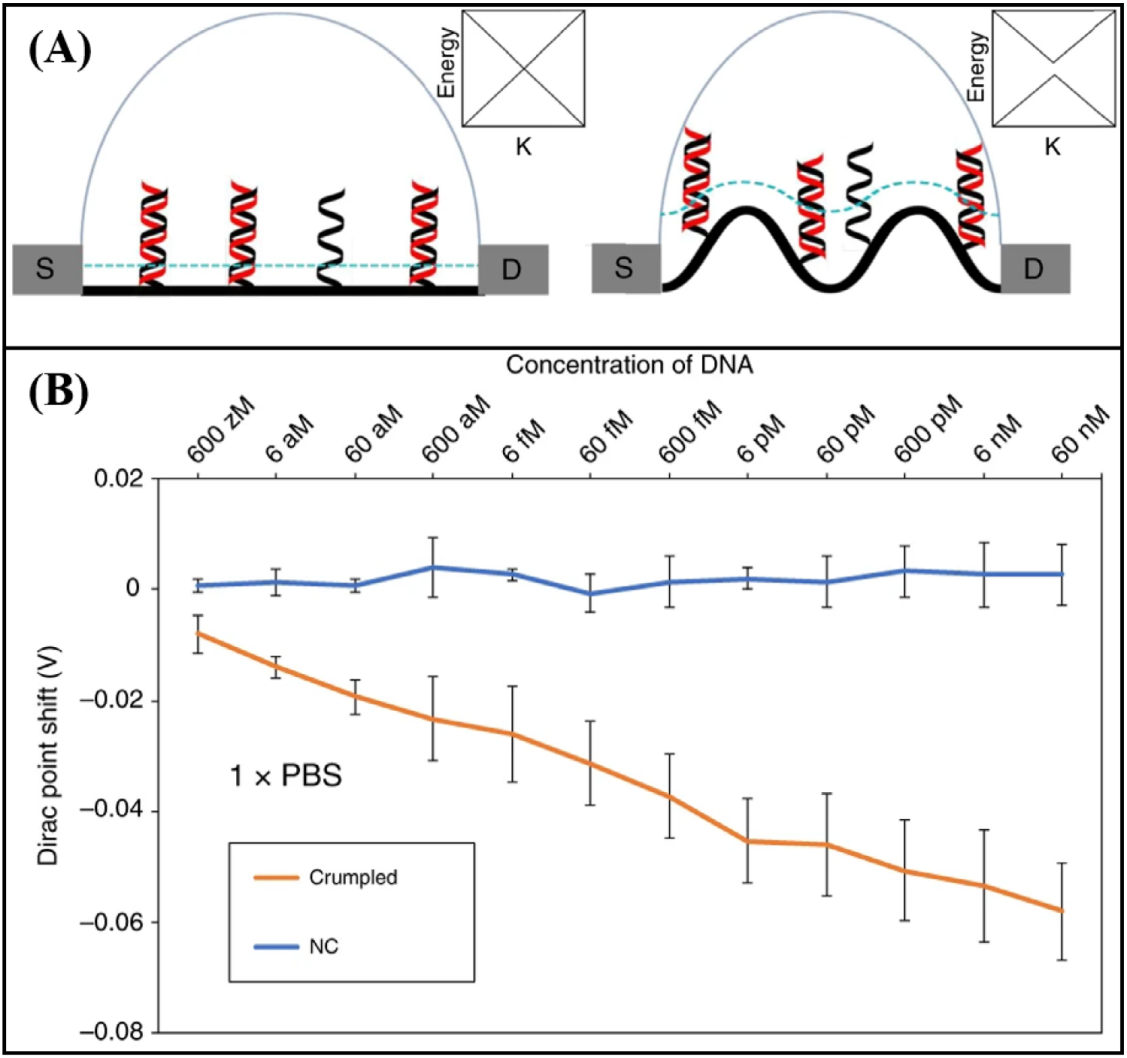
A) Schematic diagram and band diagram of the original graphene FET biosensor and the deformed graphene FET biosensor. B) deformed graphene FET biosensor for nucleic acid detection.^[^ ^187^ ^]^ Copyright 2020, Springer Nature.

Graphene‐based FET biosensors, exhibit a broad spectrum of applicability within the domain of nucleic acid detection. Gao et al.^[^ ^81^ ^]^ engineered a functionalized graphene FET sensor capable of detecting RNA. To facilitate the activation process, poly‐L‐lysine (PLL) was electrostatically adsorbed onto the graphene surface. The PLL serves as a coupling agent, connecting graphene to probe DNA. Upon specific recognition between the DNA probe and RNA, an N‐doping effect occurs in the graphene device, leading to electrostatic charging in the graphene channel and a leftward shift of the Dirac point. By monitoring the varying Dirac voltages generated from different concentrations of the target RNA, ultra‐sensitive detection of breast cancer miRNA (miR‐21, miR‐125, miR‐191, and miR‐4732) (**Figure** **11**A) and SARS‐CoV‐2 RNA (Figure 11B) was achieved, with a detection limit as low as 1 fM. In addition, Hwang et al.^[^ ^187^ ^]^ developed a deformed graphene FET sensor specifically designed for the detection of nucleic acids (**Figure** **12**A). This sensor demonstrated an impressive detection limit, with a LOD reaching down to 600zM for DNA (Figure 12B). The deformation and wrinkling of the graphene, induced by processes such as annealing, lead to nanoscale bending and deformation. These structural changes increase the Debye length in ionic solutions, effectively reducing the screening effect on DNA/RNA molecules. As a result, when compared to traditional flat graphene‐based FET biosensors, the deformed graphene sensor exhibits a significant improvement in sensitivity.

**Figure 13:**
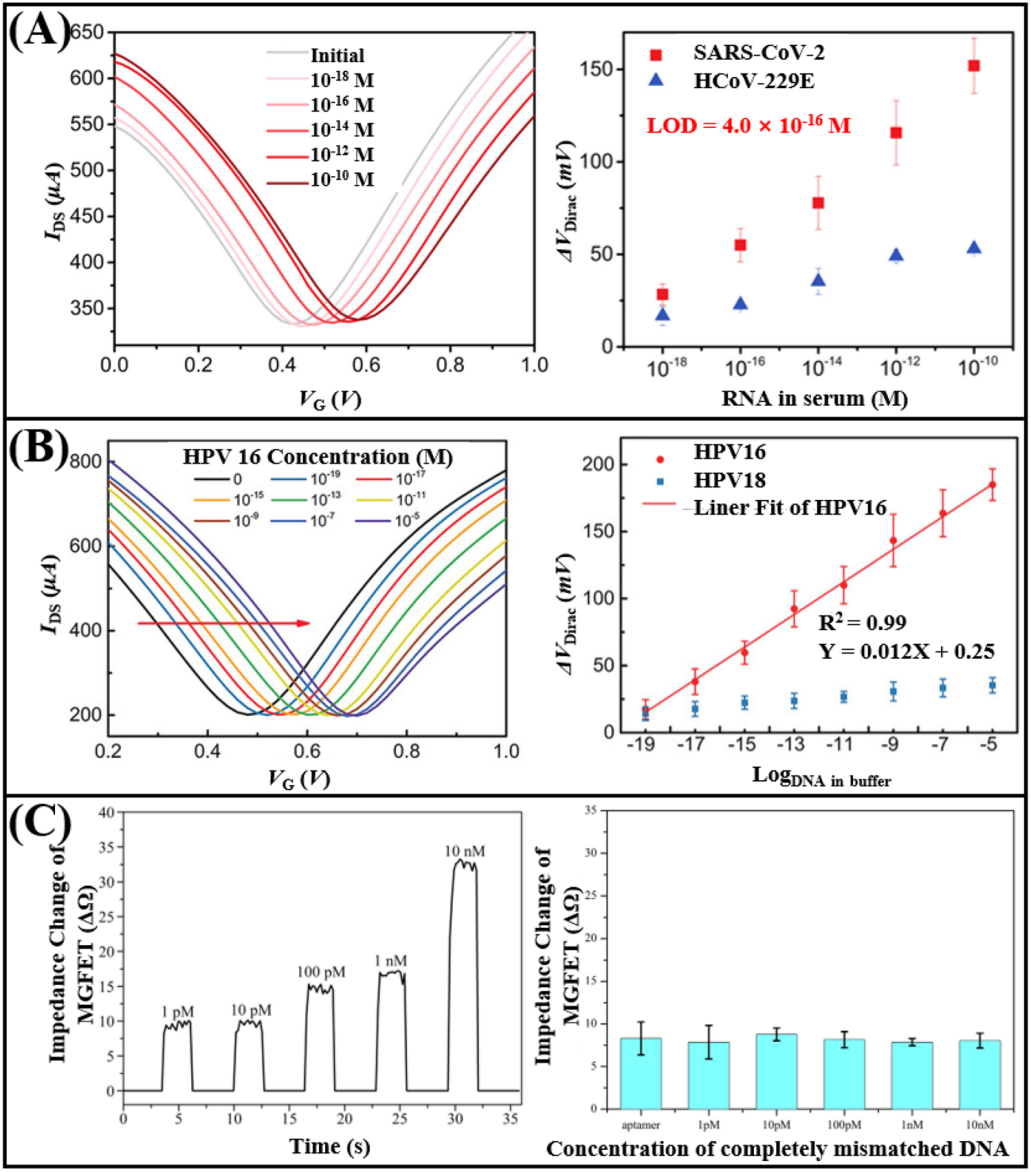
A) Liquid‐gate graphene FET biosensor for the detection of SARS‐CoV‐2 RNA.^[^ ^60^ ^]^ Copyright 2022, American Chemical Society. B) Liquid gate graphene FET biosensor for detection of HPV16 DNA.^[^ ^58^ ^]^ Copyright 2023, Wiley‐VCH. C) Magnetic graphene FET biosensor for detecting single‐strand DNA.^[^ ^85^ ^]^ Copyright 2019, Springer Nature.

In addition to the aforementioned nucleic acid detection sensors, advancements have also been made in non‐amplification and marker‐free detection methods. The integration of Clustered Regularly Interspaced Short Palindromic Repeats (CRISPR) associated nuclease (Cas) technology with graphene FET biosensing technology facilitates the detection of DNA or RNA at aM levels. Yu et al.^[^ ^60^ ^]^ designed a solution‐gated graphene FET biosensor based on the CRISPR‐Cas13a system for the detection of SARS‐CoV‐2 RNA. The CRISPR‐Cas13a ribonucleoprotein complex, or Cas13a RNPs, is made up of CRISPR RNA and Cas13a efficacious protein. By altering Cas13a RNPs at the gate, target RNA can be selectively recognized. Cas13a RNPs bind more target RNA molecules as the concentration of target RNA rises because electronegative RNA bound to the sensing surface can increase the electronegativity of the gate. This causes the transfer curve to move toward the corrected gate voltage, enabling quantitative detection of target RNA through changes in Dirac voltage. The LOD was 400 aM in serum, and the reaction time was roughly 10 min (**Figure** **13**A). CRISPR‐Cas12a functionalized solution‐gated graphene transistors were employed by Zhang et al.^[^ ^58^ ^]^ to detect HPV16 DNA without an amplification process. The sensor can detect up to 8.3 aM, and it can finish the process in 20 min (Figure 13B). The above results show the great potential of CRISPR‐based graphene FET in nucleic acid detection.

Xenes FET, employed for the detection of nucleic acids, has also achieved considerable advancements in the realm of real‐time monitoring. A magnetic graphene field effect transistor biosensor with a detection limit of 1 pM and a reaction time of less than a minute was created by Sun et al (Figure 13C).^[^ ^85^ ^]^ to detect single‐stranded DNA. Magnetic nanobeads are used to magnetically label the single‐strand DNA that needs to be detected. The impedance of the biosensor displays periodic oscillations in a periodic magnetic field when the probe is coupled to the DNA tagged by the magnetic nanobeads; the amplitude of these oscillations is correlated with the concentration of single‐strand DNA. This magnetic induction technology offers an efficient approach for the real‐time monitoring of nucleic acids, thereby establishing a crucial foundation for future real‐time DNA/RNA monitoring.

#### 4.1.2 Graphdiyne

**Figure 14:**
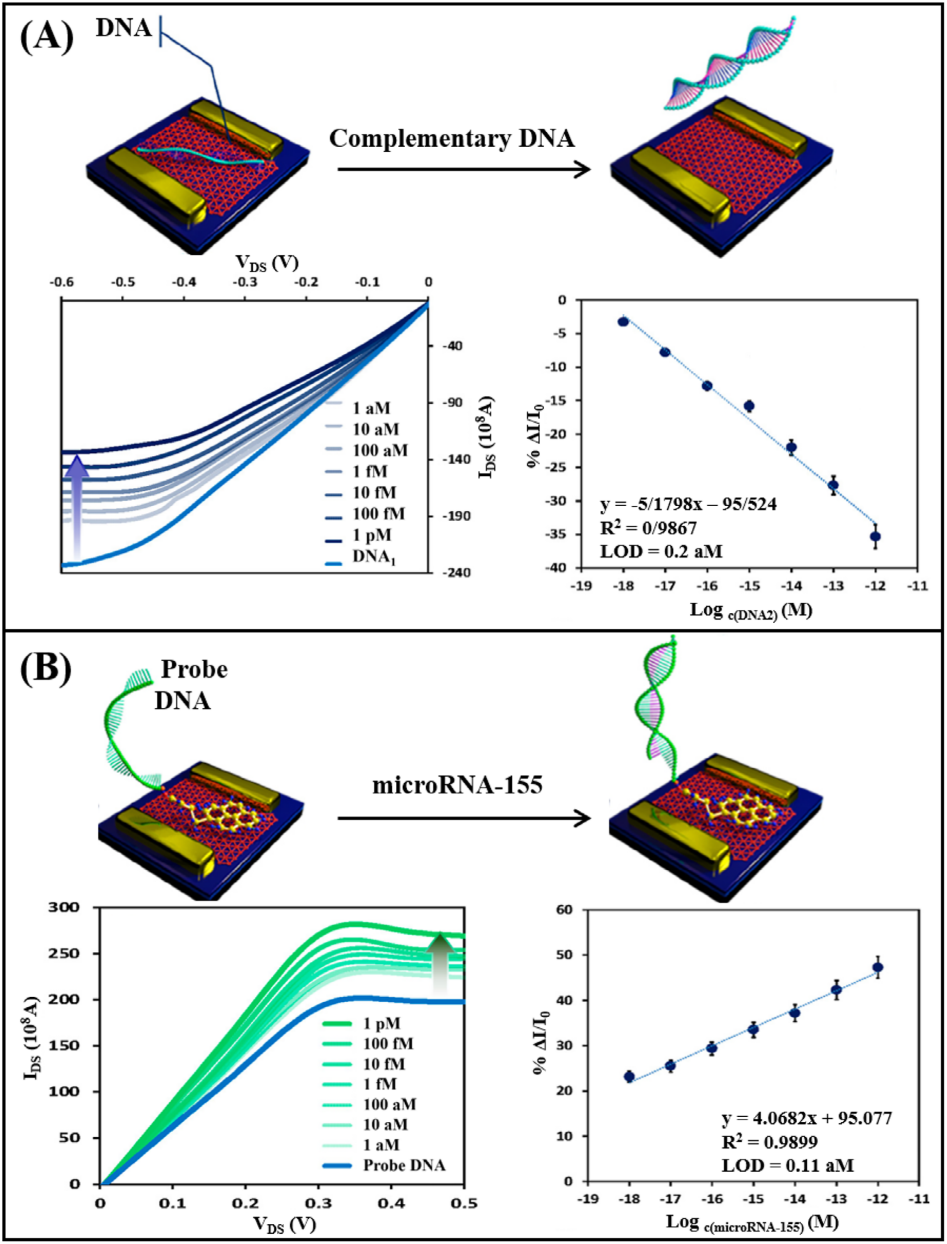
A) Graphidiyne FET biosensor for DNA detection based on the principle of electrostatic adsorption. B) Graphidiyne FET biosensor for micorRNA‐155 detection.^[^ ^56^ ^]^ Copyright 2022, American Chemical Society.

Graphdiyne, as an intrinsic semiconductor with a natural bandgap, is emerging as a novel Xenes in the realm of DNA sensor research due to its large specific surface area and excellent chemical stability. Ghafary^[^ ^56^ ^]^ et al. developed a graphdiyne FET for detecting DNA strands and microRNA‐155, employing distinct detection mechanisms for each nucleic acid. The detection of DNA strands is based on electrostatic adsorption, where the phosphate groups of probe DNA adsorb onto the graphdiyne surface, increasing the negative charge and inducing a positive charge on the graphdiyne surface, leading to an increase in channel current. Upon hybridization with target DNA, the hybridized DNA desorbs from the graphdiyne surface, causing the channel current to gradually decrease (**Figure** **14**A). In contrast, for the detection of microRNA‐155, when probe DNA hybridizes with the target microRNA on the surface, the negative charge of the microRNA is added to the probe DNA, thus forming more holes on the channel surface, leading to an increase in channel current (Figure 14B). Based on these two different principles, graphdiyne FET achieved the detection of DNA strands and microRNA‐155, with LOD of 0.2 aM and 0.11 aM, respectively. In summary, by leveraging the exceptional hole transfer characteristics of graphdiyne as the p‐type channel in FET, a new approach for nucleic acid sensors has been provided.

#### 4.1.3 Other Xenes

**Figure 15:**
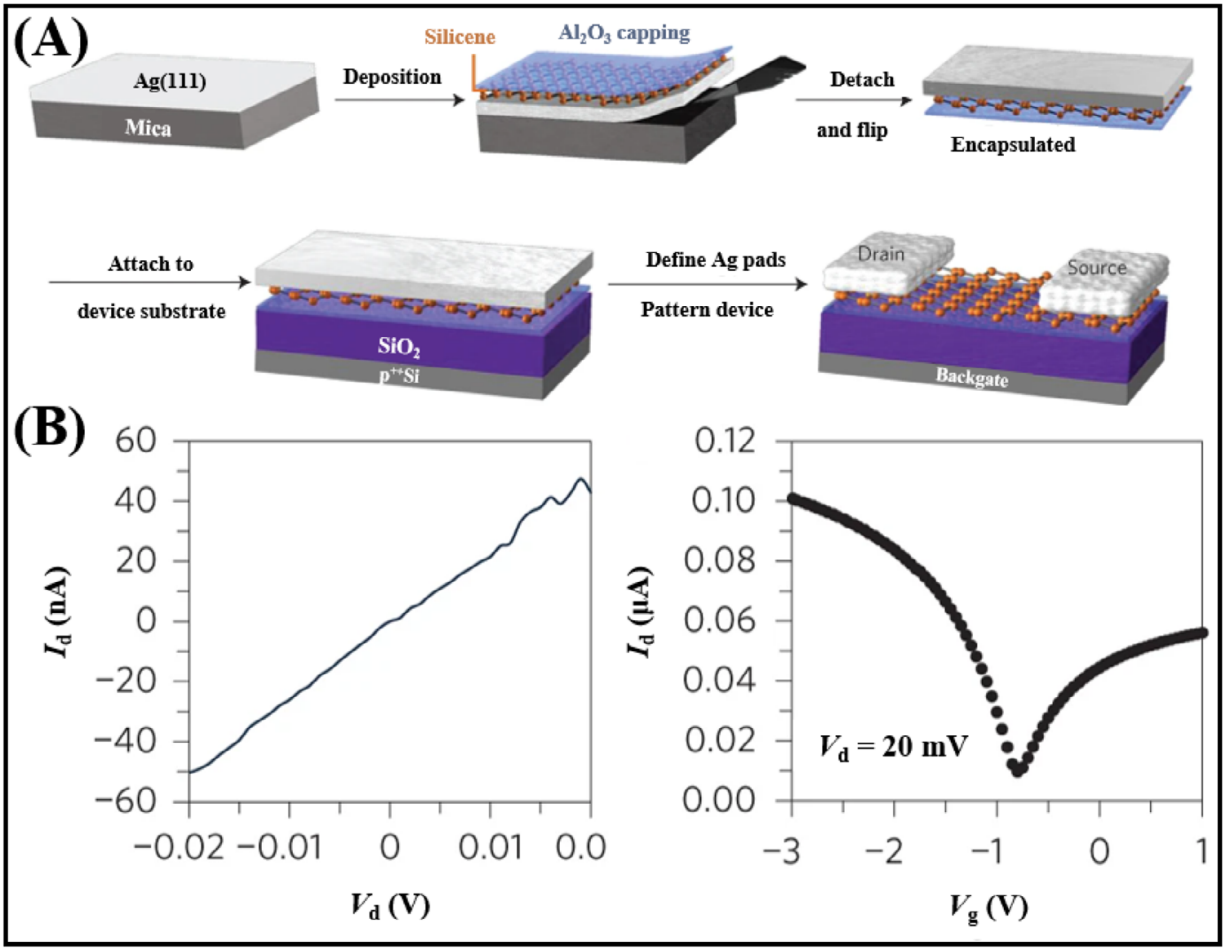
A) Schematic diagram of the growth‐transfer‐fabrication process for preparing silicene FET. B) Electrical properties of silene FET.^[^ ^141^ ^]^ Copyright 2015, Springer Nature.

In fact, aside from graphene and graphdiyne, other Xenes have not yet seen concrete applications in the domain of nucleic acid detection. However, the realization of nucleic acid detection using silicene FET is highly promising. First, silicene FET have been successfully developed. In 2015, Li^[^ ^141^ ^]^ et al. successfully developed silicene FET capable of operating at room temperature. These silicene FET were prepared through a growth‐transfer‐fabrication process (**Figure** **15**A). The silicene FET fabricated by this method exhibit Ohmic contact under ambient conditions, and their transfer characteristic curves align with the expected ambipolar electron–hole symmetry, with a carrier mobility of ≈100 cm^2^V^−1^s^−1^ (Figure 15B). Second, FET based on 1D silicon nanowires (SiNW) have been utilized for nucleic acid detection. For instance, Gao^[^ ^199^ ^]^ et al. employed SiNW FET, which demonstrated a highly sensitive detection of DNA with a LOD as low as 1 fM. These groundbreaking research outcomes not only provide a solid scientific foundation for the application of silicene FET in nucleic acid detection but also pave the way for new possibilities in the future development of silicene FET technology. Guo et al.^[^ ^200^ ^]^ conducted a theoretical exploration of the potential of tellurene for detecting DNA/RNA nucleobases using first‐principles calculations. They utilized DFT to examine the interactions between tellurene and nucleobases, which include adenine (A), thymine (T), guanine (G), cytosine (C), and uracil (U). Through precise calculations of adsorption energies, the research team found significant differences in the adsorption capacities of tellurene for the different nucleobases, with the highest adsorption energy being for G, indicating the highest sensitivity of tellurene to G. This characteristic implies that tellurene monolayers may have a significant role in DNA sequencing technology.

This section summarizes the representative applications of Xenes FET nucleic acid sensors, and **Table** **2** summarizes the sensing performance of Xenes FET nucleic acid sensors.

**Table 2:** Sensing Performance of Xenes FET Nucleic Acid Sensors.

Channel material	Analyte	Receptor	LOD	Condition	Refs.
Graphene	miRNA	DNA probe	1 fM	serum	[81]
Graphene	SARS‐CoV‐2 RNA	DNA probe	1 fM	throat swab solution	[81]
Graphene	miRNA let‐7b	DNA probe	600 zM	1 × PBS	[187]
Graphene	miRNA let‐7b	DNA probe	20 aM	human serum	[187]
Graphene	SARS‐CoV‐2 RNA	CRISPR‐Cas13a	400 aM	serum	[60]
Graphene	HPV16 DNA	CRISPR‐Cas12a	8.3 aM	0.1 × PBS‐RNase free buffer with 2 mm MgCl_2_	[58]
Graphene	magnetic nanobeads/DNA	DNA probe	1 pM	PBS	[85]
Graphene	DNA	DNA probe	25 aM	10 mM PB	[84]
Graphene	DNA	DNA probe	1 fM	PBS	[55]
Graphene	miRNA‐4484	DNA probe	10 fM	1 × PBS	[201]
Graphene	RNA	DNA probe	0.1 fg/mL	throat swab solution	[202]
Graphene	SARS‐CoV‐2 RNA	phosphorodiamidate morpholino oligos	0.37 fM	PBS	[203]
Graphene	SARS‐CoV‐2 RNA	phosphorodiamidate morpholino oligos	2.29 fM	throat swab solution	[203]
Graphene	SARS‐CoV‐2 RNA	phosphorodiamidate morpholino oligos	3.99 fM	serum	[203]
Graphene	SARS‐CoV‐2 RNA	DNA	0.03 copy/µL	throat swab solution	[204]
Graphdiyne	DNA strands	DNA	0.2 aM	PBS (0.01 M, pH 7.4)	[56]
Graphdiyne	microRNA‐155	DNA probe	0.11 aM	PBS (0.01 M, pH 7.4)	[56]

### 4.2 Protein Sensors

Proteins are pivotal functional molecules that play a crucial role in monitoring enzyme activity,^[^ ^205^ ^]^ aiding in the early diagnosis of diseases,^[^ ^206^, ^207^ ^]^ and participating in various biological processes. The quantitative detection of proteins is an essential method for evaluating protein expression levels, which holds significant importance for biomedical research.^[^ ^28^ ^]^

#### 4.2.1 Graphene

Xenes FET has shown advancements in the sensitive detection of various protein concentrations. For instance, Seo et al.^[^ ^82^ ^]^ utilized graphene as a channel material to fabricate FETs, thereby achieving highly sensitive detection of SARS‐CoV‐2 spike proteins. Initially, the coupling agent 1‐pyrenebutyric acid N‐hydroxysuccinimide ester (PBASE) was adsorbed onto the graphene surface via π‐π stacking interactions. Subsequently, PBASE formed a covalent linkage with the amino groups present on the SARS‐CoV‐2 spike antibody, resulting in the immobilization of the antibody onto the graphene surface and thus functionalizing it. Finally, the high‐sensitivity detection of the SARS‐CoV‐2 spike protein was achieved through the specific binding of the SARS‐CoV‐2 spike antibody to the SARS‐CoV‐2 spike protein. The sensor exhibits strong selectivity, a wide detection range, and a low detection limit of 1 fg/mL in PBS buffer solution.

**Figure 16:**
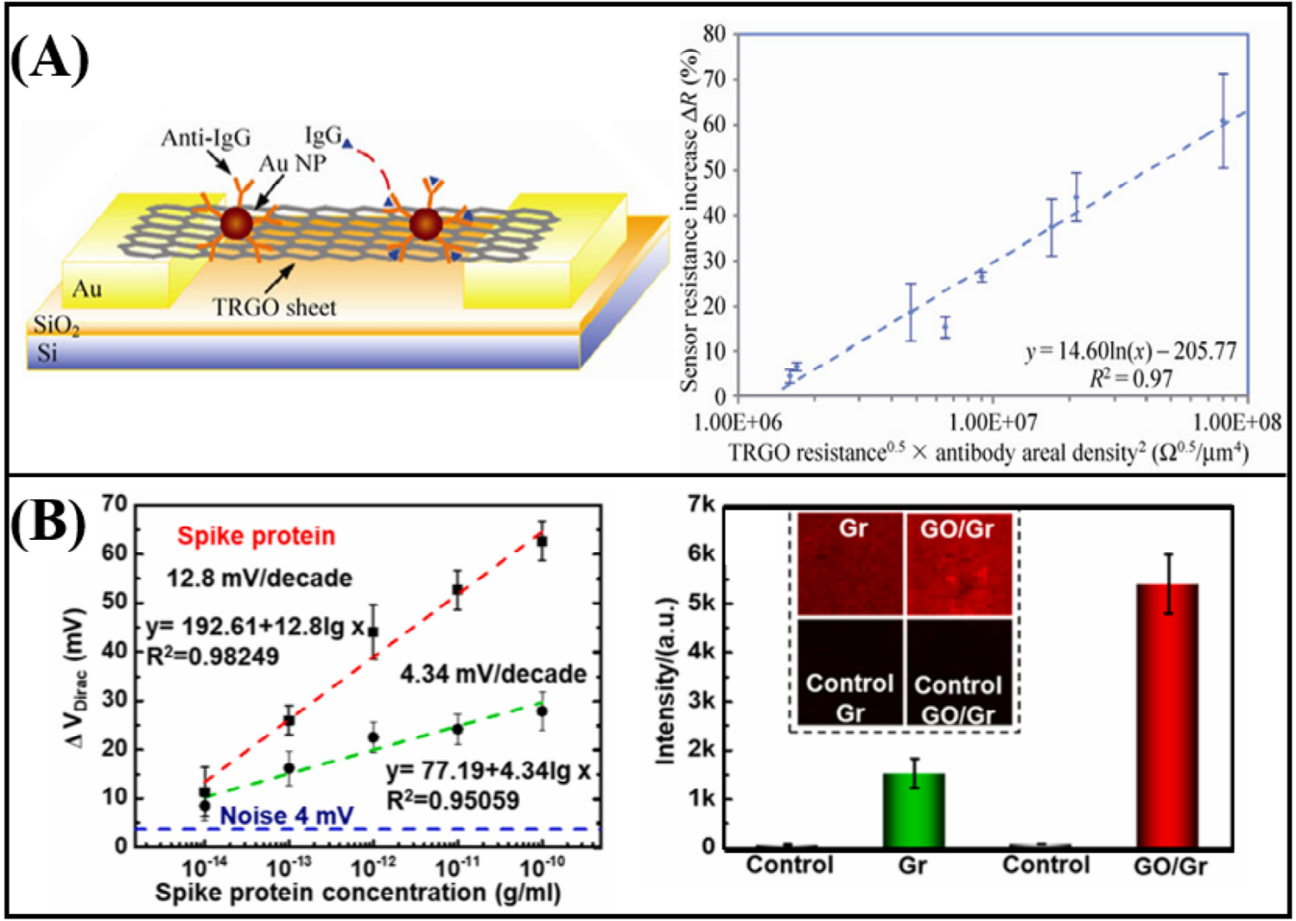
A) Thermal reduction graphene oxide FET for the detection of immunoglobulin G/IgG.^[^ ^208^ ^]^ Copyright 2011, Springer Nature. B) Graphene oxide – Graphene van der Waals heterostructure FET for SARS‐CoV‐2 protein detection.^[^ ^62^ ^]^ Copyright 2022, Elsevier.

With the advancements in graphene FET biosensors, a diverse range of graphene‐derived materials have also been integrated into protein detection technologies. To detect immunoglobulin G/IgG, Mao et al.^[^ ^208^ ^]^ created a field effect transistor based on thermally reduced graphene oxide. Anti‐immunoglobulin G/Anti‐IgG was coupled to thermo‐reduced graphene oxide using gold nanoparticles as coupling agents. Protein detection was accomplished by selective binding of antigen and antibody, with a detection limit of roughly 0.2 ng mL^−1^ (**Figure** **16**A). Gao et al.^[^ ^62^ ^]^ constructed a graphene oxide‐graphene Van der Waals heterostructure FET to detect SARS‐CoV‐2 protein. The sensor boasts a detection time of under 20 min, with a dynamic range spanning from 10 fg mL^−1^ to 100 pg mL^−1^ (Figure 16B). Notably, the detection threshold is as low as 8 fg mL^−1^, which surpasses the sensitivity of conventional graphene FET sensors by approximately threefold. This enhanced performance is primarily ascribed to the increased SARS‐CoV‐2 capture antibody immobilization density brought about by the addition of the GO layer to the graphene surface.

Graphene FET and AI have been combined in several research thus far to detect biomolecules. Previous graphene nanogrid biosensors had detected hepatitis B surface antigen (Hep‐B) down to 0.1 fM in buffer solutions, but not in physiological analytes. Basu et al.^[^ ^209^ ^]^ successfully accomplished the highly sensitive detection of Hep‐B in serum by integrating graphene nanogrid FET biosensors with artificial neural network technology. Initially, the variations in *I*_DS_ of the graphene FET biosensor at different concentrations of Hep‐B were measured, supplemented by capacitive mode measurements to extract the resonant frequency as a supportive parameter. Utilizing these parameters, an artificial neural network was trained to identify and quantify Hep‐B in serum. The network employed a fully connected feedforward architecture, with linear activation for the input and output layers, and SReLu activation functions for the hidden layers, enhancing the capability to learn complex nonlinear relationships. This approach achieved a LOD for Hep‐B in serum as low as 0.1 fM. Hemamalini et al.^[^ ^210^ ^]^ integrated graphene FET biosensors into system equipment for the detection of COVID‐19. Biomarkers are identified using graphene FET through the interaction of the biosensing component with the analyte. They also implemented AI‐based X‐ray images to detect hidden features. Finally, they integrated a multi‐layer perceptron classifier for multi‐class disease classification. A small number of experiments have integrated AI and graphene FET sensors for biomolecule identification, with encouraging outcomes.

#### 4.2.2 Graphdiyne

Owing to its excellent chemical stability and biocompatibility, graphdiyne is regarded as an ideal choice for the development of high‐performance immunosensors. Ghafary^[^ ^56^ ^]^ and colleagues developed a Graphdiyne FET immunosensor that detected a variety of proteins, all with very high sensitivity. The LODs for CA19‐9 antigen, CA15‐3 antigen, and COVID‐19 antigen were 0.04 pU mL^−1^, 0.043 pU mL^−1^, and 0.003 fg mL^−1^, respectively. The sensitivity of CA19‐9 antigen and CA15‐3 antigen detection matches the clinical diagnostic requirements and can be used for early cancer diagnosis. The detection sensitivity of the COVID‐19 antigen meets the needs for rapid and accurate diagnosis. In summary, Graphdiyne FET immunosensors show great potential in the detection of disease biomarkers, and their high sensitivity, selectivity, and rapid response make them promising for future biological detection and disease diagnosis.

#### 4.2.3 BP

**Figure 17:**
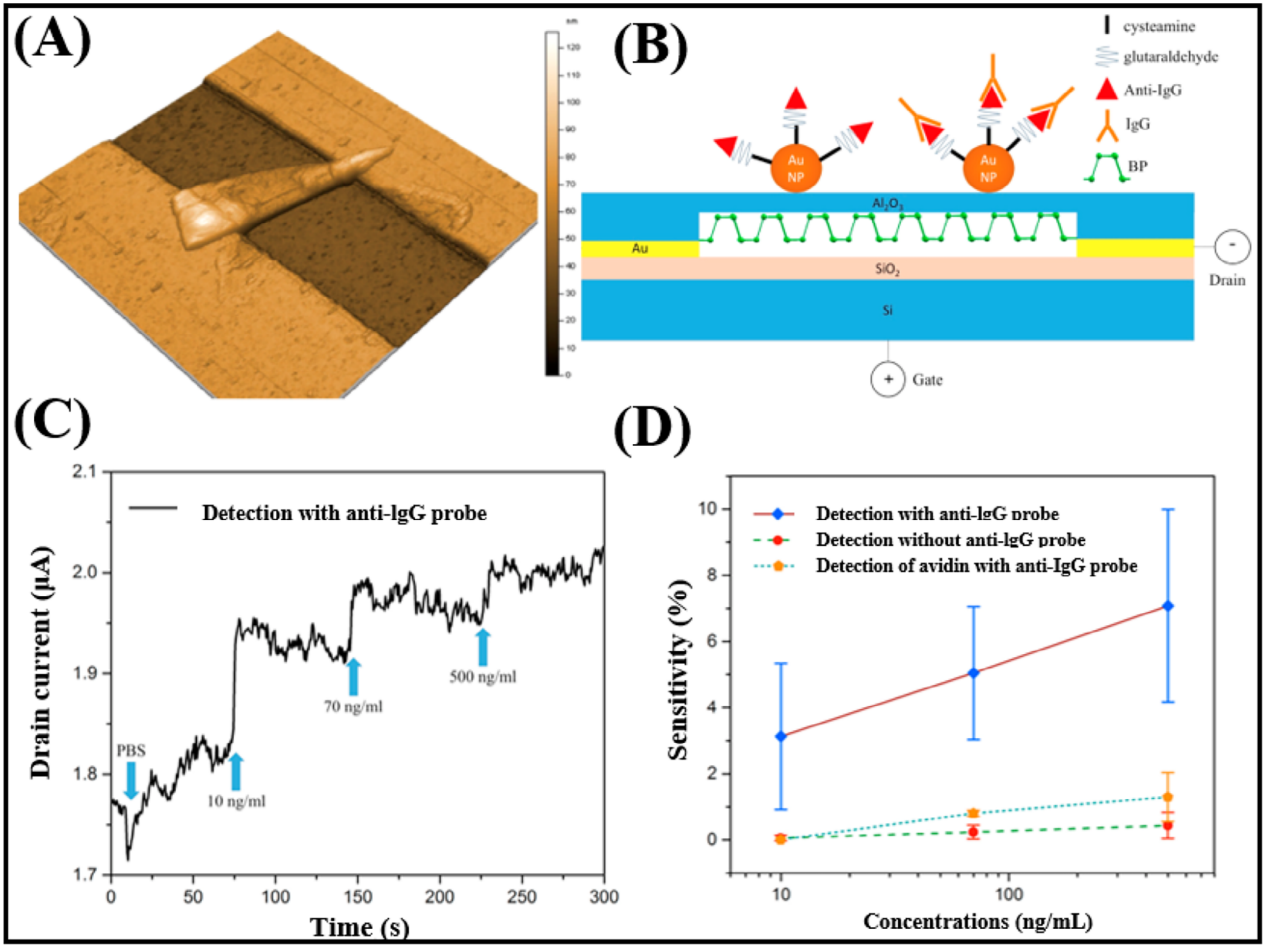
A) 3D view of BP FET. B) Structure diagram of BP FET biosensor. C) Real‐time response curve of BP FET biosensor to different concentrations of IgG. D) Sensitivity and specificity of BP FET biosensor.^[^ ^61^ ^]^ Copyright 2017, Elsevier.

In addition to graphene FET and graphdyien FET, BP FET also possess specific applications within the domain of protein detection. Chen et al.^[^ ^61^ ^]^ prepared FET based on BP nanosheets and used them to detect human immunoglobulin G (IgG) (**Figure** **17**A). BP nanosheets, derived from mechanical exfoliation, serve as channel materials in FET. However, due to the propensity of BP to oxidize when exposed to air,^[^ ^42^ ^]^ Al_2_O_3_ thin films are employed as surface passivation layers. These dielectric layers effectively shield the BP from atmospheric oxygen and moisture. The gold nanoparticles were deposited onto the surface of BP via a sputtering technique, which involved surface functionalization. Subsequently, antibodies were covalently attached to these gold nanoparticles (Figure 17B). The interaction between the antigen and antibody resulted in a modification of the gate potential, consequently altering the drain current (Figure 17C). The fabricated black phosphorous biosensor exhibited exceptional sensitivity and specificity toward human IgG, with a detection threshold as low as 10ng mL^−1^ (Figure 17D). This investigation conclusively illustrates the substantial potential of BP nanosheets to serve as FET biosensor channels, enabling highly sensitive and selective detection of biomarkers.

To date, significant advancements have been achieved in the development of FET protein sensors utilizing various single‐element 2D materials and functionalization techniques. **Table** **3** provides a summary of the sensing performance of the Xenes FET protein sensor.

**Table 3:** Sensing Performance of Xenes FET Protein Sensors.

Channel material	Analyte	Receptor	LOD	Condition	Refs.
Graphene	SARS‐CoV‐2 spike protein	SARS‐CoV‐2 spike antibody	1.6 × 10^1^ pfu/mL	culture medium	[82]
Graphene	SARS‐CoV‐2 spike protein	SARS‐CoV‐2 spike antibody	1 fg/ml	PBS	[82]
Graphene	SARS‐CoV‐2 spike protein	SARS‐CoV‐2 spike antibody	2.42 × 10^2^ copies/mL	clinical samples	[82]
Graphene	SARS CoV‐2 spike protein	antibody	1 aM	0.1 × PBS	[211]
Graphene	Anti‐diuretic hormone	aptamer	3.55 ag/mL	10 mm PBS	[212]
Graphene	p53 protein	p53 antigen	10 nM	1 × PBS	[213]
Graphene	t‐Tau protein	antibody	10fg/mL	1 × PBS	[214]
Graphene	thrombin	aptamer	1 fM	0.1 × PBS	[207]
Graphene	prostate specific antigen	antibody	1 pg/mL	serum	[215]
Graphene	prostate specific antigen	antibody	1 nM	1 × PBS	[216]
Graphene	IL‐6	antibody	4 aM	1 × PBS	[211]
Graphene	IL‐6	aptamer	618 fM	1 × PBS	[217]
Graphene	Zika viral nonstructural protein 1	antibody	0.45 nM	1 × PBS	[218]
Graphene	insulin	aptamer	766 fM	1 × PBS	[217]
Graphene	insulin	aptamer	35 pM	1 × PBS	[219]
Graphene nanogrid	Hep‐B surface antigen	anti‐Hep‐B monoclonal antibody	0.1 fM	serum	[209]
Thermally reduced graphene oxide	Immunoglobulin G/IgG	anti‐Immunoglobulin G/anti‐IgG	0.2 ng/mL	PBS (pH 7.4, ×1)	[208]
Graphene oxide‐graphene Van der Waals heterostructure	SARS‐CoV‐2 protein	SARS‐CoV‐2 antibody	8 fg/mL	1 × PBS	[62]
Reduced graphene oxide	Aβ protein	6E10 antibody	1 pg/mL	0.1 mM PBS	[220]
Reduced graphene oxide	Aβ_1‐42_ protein	Aβ‐specific antibody	0.1pg/mL	PBS/Human plasma/aCSF	[220]
Reduced graphene oxide	t‐Tau protein	Tau‐specific antibody	0.1pg/mL	PBS/Human plasma/aCSF	[220]
Graphdiyne	CA19‐9 antigen	CA19‐9 antibody	0.04 pU/mL	PBS	[56]
Graphdiyne	CA15‐3 antigen	aptamer	0.043 pU/mL	PBS	[56]
Graphdiyne	COVID‐19 antigen	COVID‐19 antibody	0.003 fg/mL	PBS	[56]
BP	human immunoglobulin G	antibody	10ng/mL	PBS	[61]

### 4.3 Small Biomolecular Sensors

Small biomolecules, characterized by an average molecular weight of less than 500 kDa, are biologically active compounds that serve as crucial biomarkers for the early diagnosis of diseases.^[^ ^28^, ^221^ ^]^ These primarily include glucose,^[^ ^151^ ^]^ dopamine,^[^ ^211^ ^]^ amino acids,^[^ ^222^ ^]^ urea^[^ ^223^ ^]^ and sialic acid.^[^ ^63^ ^]^ At present, most drugs are small subclasses of drugs, and the basic component units of biological macromolecules such as proteins and nucleic acids are also small molecules. Therefore, the detection of small biological molecules is of great significance.

#### 4.3.1 Graphene

**Figure 18:**
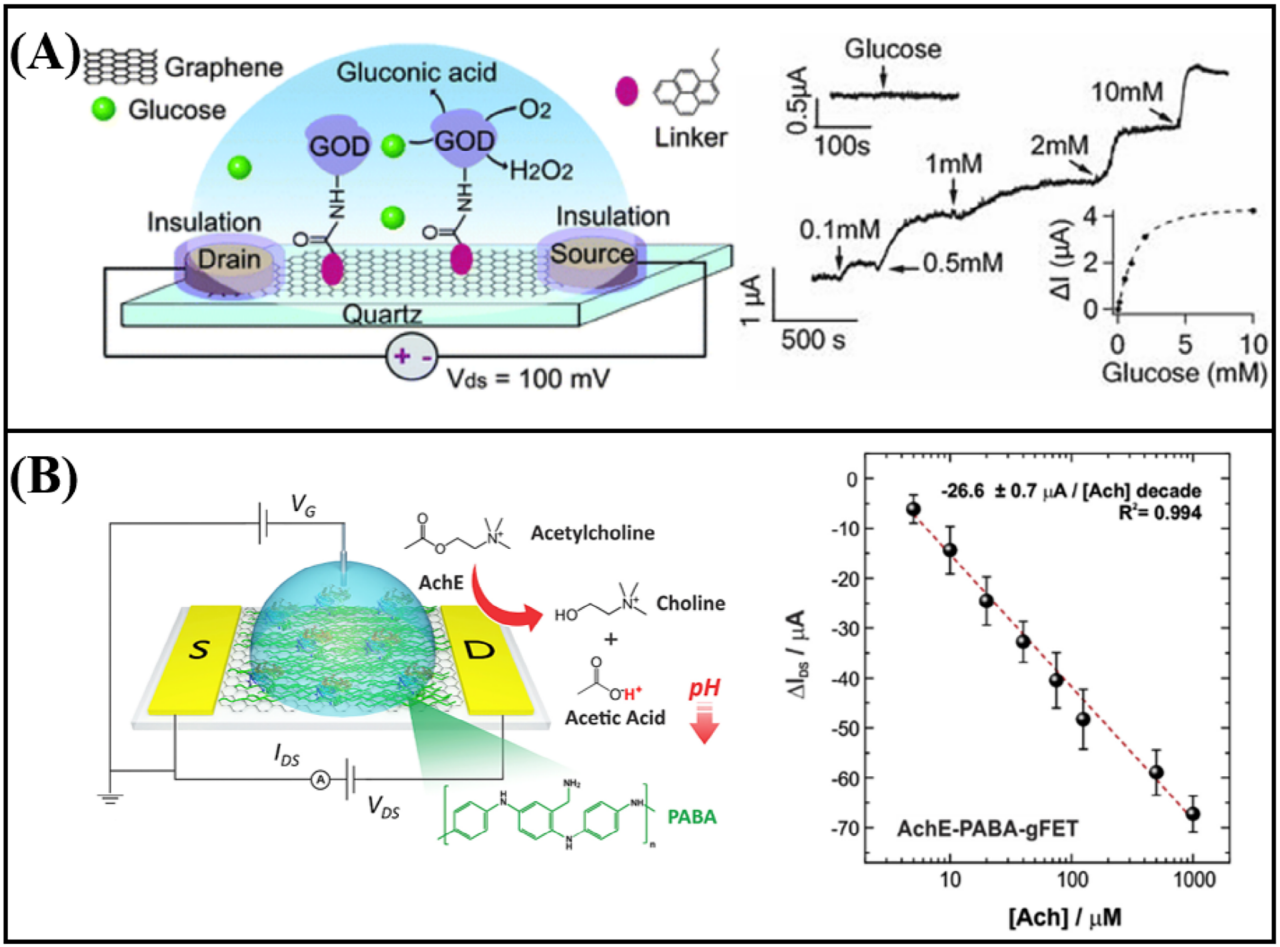
A) Graphene FET biosensor for the detection of glucose.^[^ ^151^ ^]^ Copyright 2010, Royal Society of Chemistry. B) Graphene FET biosensor for the detection of acetylcholine.^[^ ^225^ ^]^ Copyright 2020, Elsevier.

Initially, the primary use of Xenes FET small biomolecular sensors was the detection of small biomolecules by the alteration of enzymes at the sensing interface. For example, Huang et al.^[^ ^151^ ^]^ modified glucose oxidase (GOD) on graphene FET to detect glucose. GOD catalyzes the oxidation of glucose in the following reaction: β‐D‐glucose + O_2_ + H_2_O → D‐glucono‐1,5‐lactone + H_2_O_2_. After the chemical reaction, the chemical doping level interface changes, resulting in a resulting in a significant change in the conductance of the graphene FET, thereby achieving the detection purpose. The LOD for the graphene FET glucose sensor can achieve 0.1 mM (**Figure** **18**A), which is competitive with that of commonly employed electrochemical sensors.^[^ ^224^ ^]^ Fenoy et al.^[^ ^225^ ^]^ have also developed an enzyme‐modified graphene FET for the detection of acetylcholine. The local pH reduction, induced by the hydrolysis catalyzed by acetylcholinesterase, results in a shift of the Dirac point of the graphene FET to a more negative value. This shift constitutes the signal transduction mechanism of the modified transistor. The biosensor, constructed using this method, exhibits a detection limit of 2.3 µM and a linear detection range from 5 to 1000 µM (Figure 18B). In addition to glucose and acetylcholine, enzyme‐modified graphene FET sensors can also facilitate the detection of small molecules such as arginine.^[^ ^222^ ^]^

**Figure 19:**
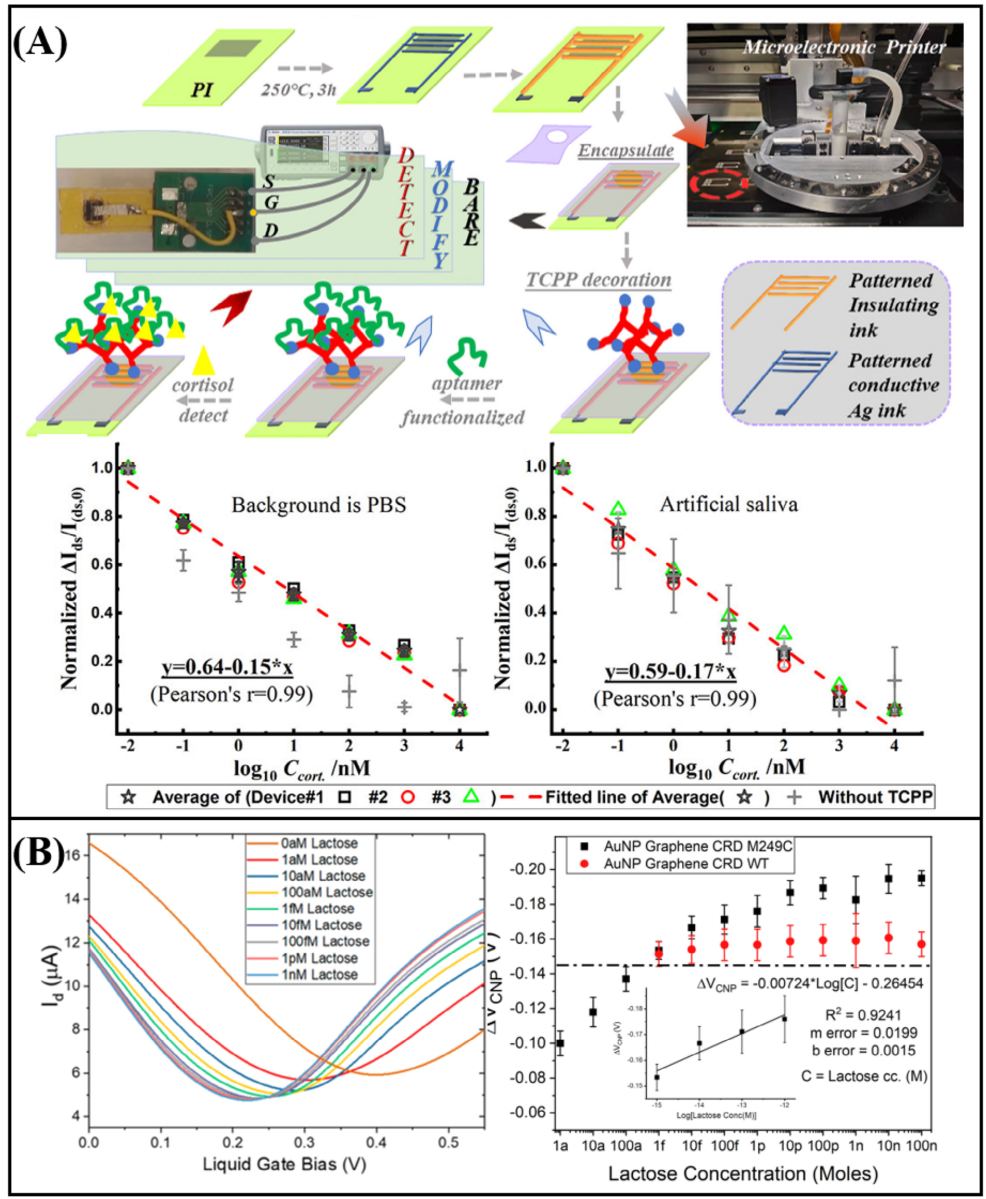
A) A liquid gate graphene FET biosensor for the detection of salivary cortisol.^[^ ^228^ ^]^ Copyright 2021, American Chemical Society. B) Graphene FET biosensor for the detection of lactose.^[^ ^229^ ^]^ Copyright 2020, Elsevier.

Although FET sensors that utilize enzyme modifications exhibit high sensitivity and selectivity, they are not without limitations. These include poor enzyme stability,^[^ ^226^ ^]^ potential damage to sensing materials,^[^ ^227^ ^]^ and an excess of active sites. In response to these challenges, non‐enzymatic FET biosensors have been developed for the sensitive detection of small biomolecules. For example, Zhang et al.^[^ ^228^ ^]^ achieved the detection of cortisol using graphene FET. The specificity and selectivity of cortisol and the interference of pH value were considered in the biological functionalization strategy. They opted for Tetrad (4‐carboxyphenyl) porphyrin (TCPP) as the aptamer probe, covalently anchoring it to the graphene FET. This modification of TCPP not only enhances the sensitivity of graphene FET to cortisol but also enables the coating of oxygen‐containing groups, thereby mitigating the response to pH fluctuations in saliva samples. The experimental outcomes indicate that the sensor is capable of detecting a linear concentration range spanning from 10 pM to 10 µM (**Figure** **19**A). Danielson^[^ ^229^ ^]^ and colleagues designed a graphene‐based FET lactose sensor that operates without the need for enzymatic activity. This sensor leverages the carbohydrate recognition domain from the human galactin‐3 protein to detect lactose, utilizing the lectin‐glycan affinity binding mechanism on graphene. The sensor boasts a detection limit of 200 aM and exhibits specificity for lactose within a dynamic range spanning from 1 fM to 1 pM (Figure 19B).

#### 4.3.2 BP

**Figure 20:**
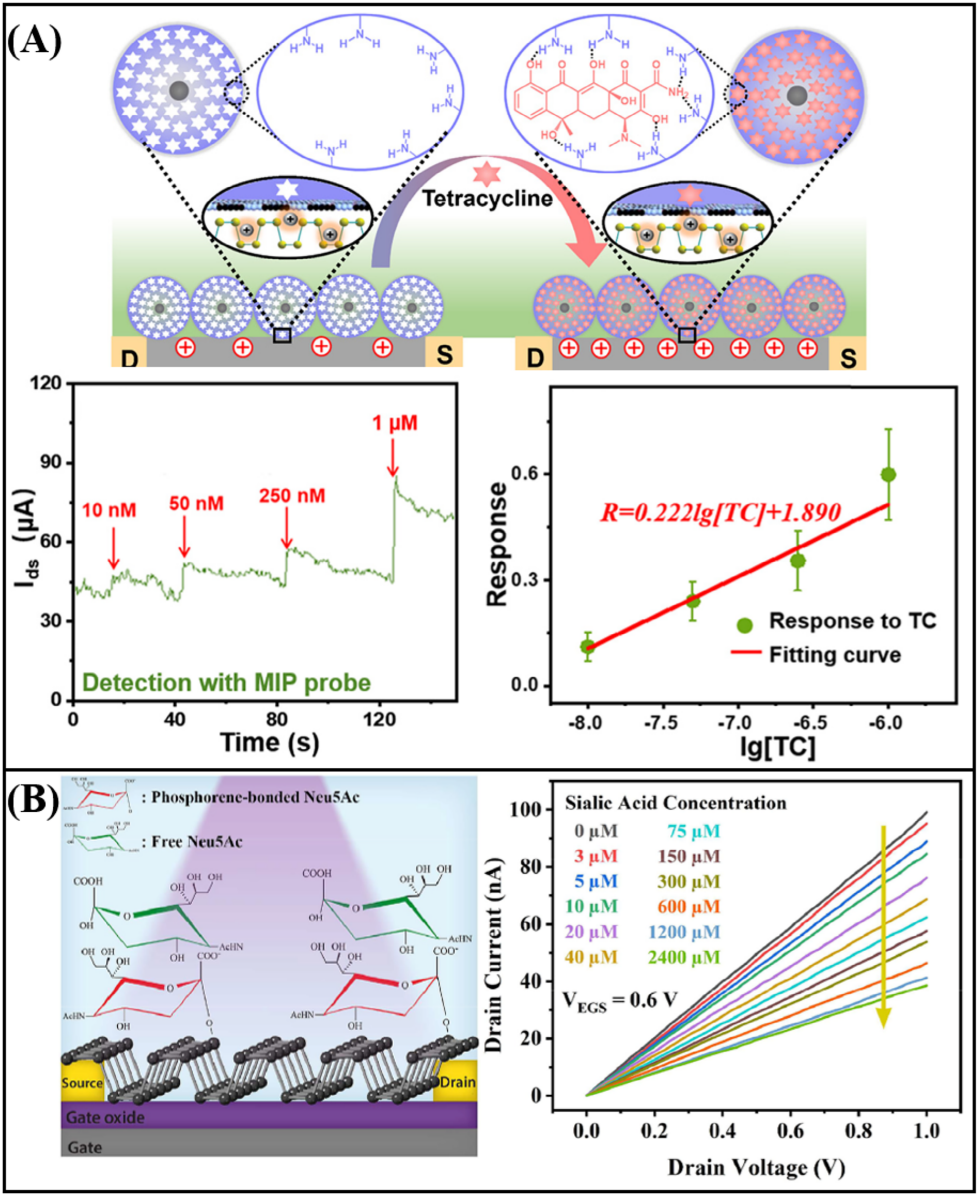
A) BP FET biosensor for the detection of antibiotics.^[^ ^57^ ^]^ Copyright 2023, American Chemical Society. B) Ph‐FET biosensor for Neu5Ac detection.^[^ ^63^ ^]^ Copyright 2023, Elsevier.

In addition, BP has also made significant advancements as a channel material for detecting small biomolecules. Chen et al.^[^ ^57^ ^]^ engineered a FET utilizing BP for the sensitive detection of tetracycline. The stability and performance of the BP FET were significantly bolstered through a novel surface engineering approach that involved Ag coordination coupled with the supramolecular passivation facilitated by melamine cyanuric acid. Incorporating a molecularly imprinted polymer as a probe for tetracycline detection, the sensor exhibited exceptional sensitivity to tetracycline, achieving a detection limit of 7.94 nM and rapid response within 6 s (**Figure** **20**A). Moreover, the sensor demonstrated high selectivity in distinguishing tetracycline from other antibiotics. Asgharian et al.^[^ ^63^ ^]^ achieved highly sensitive detection of N‐acetylneuraminic acid (Neu5Ac) based on the molecule‐induced n‐type behavior of phosphorene‐based FET (Ph‐FET). Under ultraviolet irradiation, Neu5Ac molecules interact with phosphorene atoms at the top of the puckered structure to form SA bonds. This reaction weakens the anti‐bonding orbitals of the P–P (P_4_) lattice and screens the trapping sites, thereby changing the electrical transport properties of the pristine Ph‐FET from p‐type to n‐type. Based on this mechanism, the Ph‐FET exhibited excellent performance in detecting Neu5Ac, with a LOD of 2.8 µM (Figure 20B).

**Table** **4** presents a summary of the recent advancements in Xenes FET small biomolecule sensors, along with their sensing performance metrics.

**Table 4:** Sensing Performance of Xenes FET Small Biomolecular Sensors.

Channel material	Analyte	Receptor	LOD	Condition	Refs.
Graphene	glucose	glucose oxidase	0.1 mM	10 mM PBS	[151]
Graphene	glutamate	glutamic dehydrogenase	5 µM	10 mM PBS	[151]
Graphene	acetylcholine	acetylcholinesterase	2.5 µM	KCl 10 mM, HEPES 0.1 mM, pH = 7	[225]
Graphene	arginine	arginase and urease	10 µM	10 mM KCl	[222]
Graphene	salivary cortisol	tetrakis(4‐carboxyphenyl) porphyrin	10 pM	PBS	[228]
Graphene	lactose	human galectin‐3	200 aM	PBS	[229]
Graphene	biotin		0.37 pM	bovine serum albumin	[227]
Reduced graphene oxide	dopamine	N/A	1 nM	0.1 M PBS	[230]
BP	tetracycline	molecularly imprinted polymers	7.94 nM	deionized water	[57]
BP	N‐acetylneuraminic acid	sialic acid	2.8 µM	PBS	[63]

### 4.4 Cell‐Based Biosensors

The cell serves as the fundamental unit of life. Monitoring cellular activity and products is crucial for the early diagnosis of diseases.^[^ ^231^ ^]^ This section provides a summary of the advancements in Xenes FET biosensors for cell detection.

#### 4.4.1 Graphene

**Figure 21:**
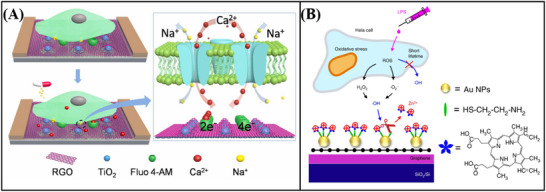
A) Renewable graphene FET biosensor for real‐time monitoring of Ca^2+^ excreted from cells.^[^ ^70^ ^]^ Copyright 2023, Elsevier. B) Graphene FET biosensor for detecting ·OH production in living cells.^[^ ^232^ ^]^ Copyright 2019, Springer Nature.

The biosensing process typically does not directly detect cells themselves, but rather analyzes them through the detection of cellular secretions or electrical potential outside the cells. Xenes FET cell sensors have achieved significant advancements in the real‐time and in situ detection of cellular secretions. For instance, Lei et al.^[^ ^70^ ^]^ employed renewable graphene FET biosensors to achieve in situ monitoring of Ca^2+^ emitted from cells. The Ca^2+^ probes molecule, Fluo4‐AM, is immobilized on the graphene sensing interface via π‐π stacking interactions. Owing to the positive charge of Ca^2+^, the binding of the probe molecule to the target analyte alters the electrical charge from 4e^−^ to 2e^−^. This transformation induces a modification in the distribution of conduction charges on the surface of the conductive channel, consequently affecting the electrical performance of the biosensor. Consequently, the concentration of Ca^2+^ in the solution can be monitored. The sensor's LOD can achieve a sensitivity of 100 pM (**Figure** **21**A). In addition, Wang et al.^[^ ^232^ ^]^ have engineered a graphene FET that is functionalized with gold nanoparticles (Au NPs) to facilitate the detection of ·OH in cellular effluents. The integration of Au NPs onto the graphene surface was achieved using cysteamine as a linker molecule. The interaction between ·OH and thiol groups leads to the release of metal ions from the graphene, inducing surface charge dedoping and eliciting a detectable current response within the FET channel. This sensor exhibits LOD for ·OH produced by viable cells up to 100 nM (Figure 21B), which is competitive with or surpasses the sensitivity of some conventional methods for free radical detection.^[^ ^64^ ^]^

Furthermore, progress has been made in the detection of extracellular potentials. Kireev et al.^[^ ^233^ ^]^ cultured cardiomyocytes and neurons directly on graphene FET arrays. These cells generate electrical signals through their natural electrophysiological activities. The cellular activity‐generated electrical signals were recorded using a multi‐channel measurement system. The recordings showed distinguishable action potentials, with a signal‐to‐noise ratio exceeding 6 for the in vitro cardiac‐like cell line. Li et al.^[^ ^234^ ^]^ characterized cellular action potentials as conductance signals using light‐activated graphene FET. This method can measure cell concentration and the expression levels of photosensitive proteins.

### 4.5 Ion Sensors

Xenes FET exhibit promising applications in ion sensing within biological environments, particularly in the realm of pH sensing. Their high sensitivity and selectivity render them ideal tools for monitoring intracellular pH variations, assessing biological responses, and evaluating environmental conditions. This provides robust technical support for biomedical research and environmental monitoring.

#### 4.5.1 Graphene

**Figure 22:**
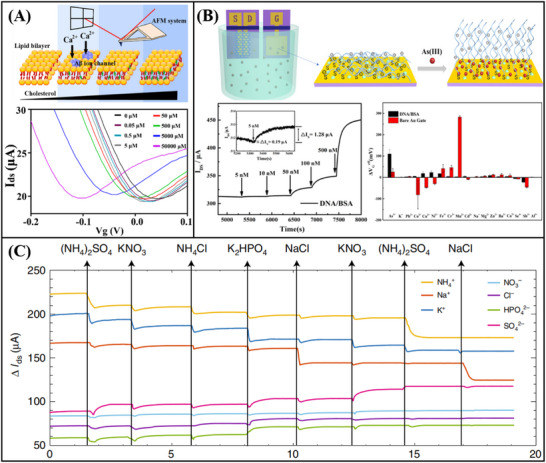
A) Graphene FET with integrated lipid bilayer are used for Ca^2+^ detection.^[^ ^65^ ^]^ Copyright 2020, American Chemical Society. B) Graphene FET for the detection of As^3+^.^[^ ^235^ ^]^ Copyright 2021, American Chemical Society. C) Real‐time response curve of graphene FET with integrated ion carrier membrane.^[^ ^236^ ^]^ Copyright 2020, Springer Nature.

Xenes FET biosensors have demonstrated significant potential in the field of ion detection, with graphene FETs emerging as a focal point of research due to their exceptional performance. Graphene surfaces are often modified by ion‐selective receptors to enable specific detection of individual ions. For instance, Gao et al.^[^ ^65^ ^]^ discovered that under the influence of 15% cholesterol, lipid bilayer can form Aβ ion channels that allow Ca^2+^ to pass through. Based on this finding, they successfully achieved highly sensitive detection of Ca^2+^ by integrating graphene FET with lipid bilayer solution, with a detection limit of 50 nM and a response time of less than 1 s (**Figure** **22**A). Furthermore, Wang et al.^[^ ^235^ ^]^ have developed a DNA‐modified extended‐gate graphene FET platform that achieves selective detection of As^3+^ in actual rice samples, with a detection limit as low as 5 nM. This selectivity is facilitated by the interaction between As^3+^ and gold, which disrupts the adsorption state of DNA, consequently leading to a redistribution of surface charge on the gate electrode. As research progresses, the detection of single ions has become increasingly inadequate for the analysis of complex samples (Figure 22B). Consequently, scientists have begun to explore the possibility of simultaneous multi‐ion detection. By integrating ionophore membranes with graphene FET arrays, simultaneous detection of various ions, including K^+^, Na^+^, NH4^+^, NO_3_^−^, SO_4_^2−^, HPO_4_^2−^, and Cl^−^, has been achieved, with detection limits all below 10 µM (Figure 22C).^[^ ^236^ ^]^ However, a major challenge in simultaneous multi‐ion detection is the mutual interference between different ions. To address this issue, Fakih et al. employed an improved Nikolskii‐Eisenman theory to account for ion interference and accurately estimate the concentrations of multiple ions. Using this method, they were able to achieve accuracies of ±0.01 and ±0.05 log concentration for cations and anions, respectively, significantly enhancing the accuracy and reliability of multi‐ion detection. Furthermore, AI‐graphene FET sensors find applications in ion detection. Xue et al.^[^ ^77^ ^]^ developed an integrated biosensor platform using graphene transistor arrays to detect ions. This platform comprises over 200 integrated sensing units, customized high‐speed readout electronics, and employs machine learning algorithms. It delivers highly sensitive, reversible, and real‐time responses to potassium, sodium, and calcium ions in complex solutions. Zhang et al.^[^ ^237^ ^]^ developed a machine learning‐assisted electrolyte grid‐controlled graphene FET for calcium ion detection. Due to the non‐uniformity of graphene FET sensors in various environments, they propose a method that combines machine learning with graphene FET. This approach not only ensures accurate results but also simplifies sensor usage by eliminating the need for calibration, thereby promoting the wider application of graphene FET sensors and similar technologies.

**Figure 23:**
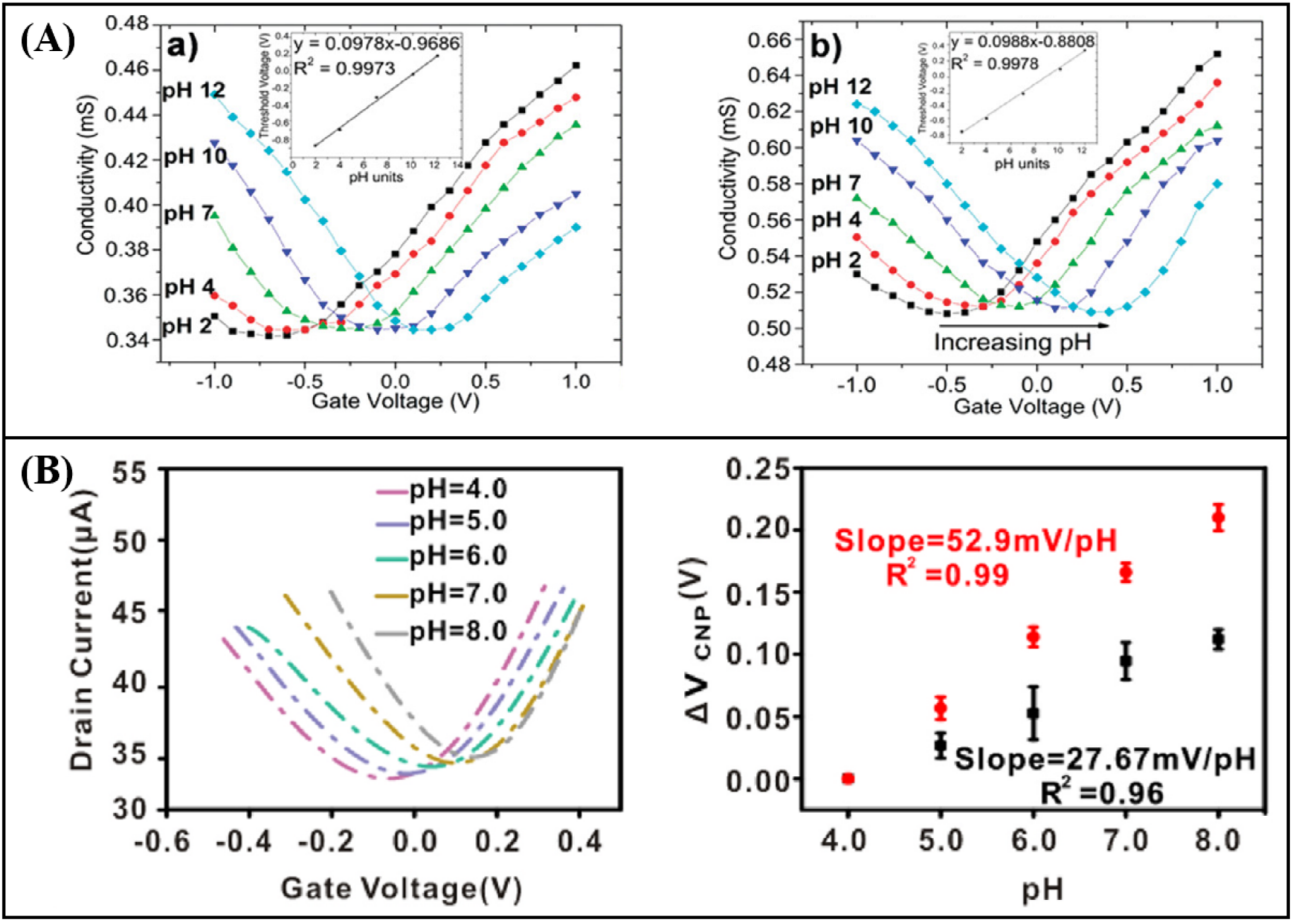
A) Variation trend of I_DS_‐V_GS_ curves of 1–2 layers graphene FET and 3–4 layers graphene FET with pH value (Illustration shows the linear correlation between pH change and Dirac voltage change.).^[^ ^238^ ^]^ Copyright 2008, American Chemical Society. B) PLL@G‐FET Sensitivity of the sensor for pH detection.^[^ ^239^ ^]^ Copyright 2023, Elsevier.

Detecting pH values is one of the key applications of ion sensors. In 2008, Ang et al.^[^ ^238^ ^]^ first proposed the solution‐gate graphene FET as a pH sensor. The fundamental principle is that the double layer at the graphene/electrolyte interface is highly sensitive to pH, allowing H_3_O^+^ or OH^−^ ions to capacitively charge the interface. As the pH value increases from 2 to 12, there is a noticeable positive shift in the Dirac voltage (**Figure** **23**A), indicating an increase in p‐type doping of graphene through the adsorption of OH^−^ ions. Both 1–2 layers graphene FET and 3–4 layers FET show a linear trend between pH changes and Dirac voltage changes (inset of Figure 23A), enabling quantitative detection of pH values. Moreover, the sensitivity of 1–2 layers graphene FET is 98 mV pH^−1^, and that of 3–4 layers graphene FET is 99 mV pH^−1^, both of which exceed the Nernst limit of 59.2 mV pH^−1^. Graphene FET platforms for pH detection also have certain applications in the biomedical field. Recently, Xiao et al.^[^ ^239^ ^]^ developed a poly‐L‐lysine modified graphene FET (PLL@G‐FET) for in situ monitoring of the external acidic environment of cancer cells. The PLL@G‐FET sensor achieved a Nernstian value of 52.9 mV pH^−1^ in phosphate‐buffered saline from pH 4.0 to 8.0 (Figure 23B).

#### 4.5.2 BP

**Figure 24:**
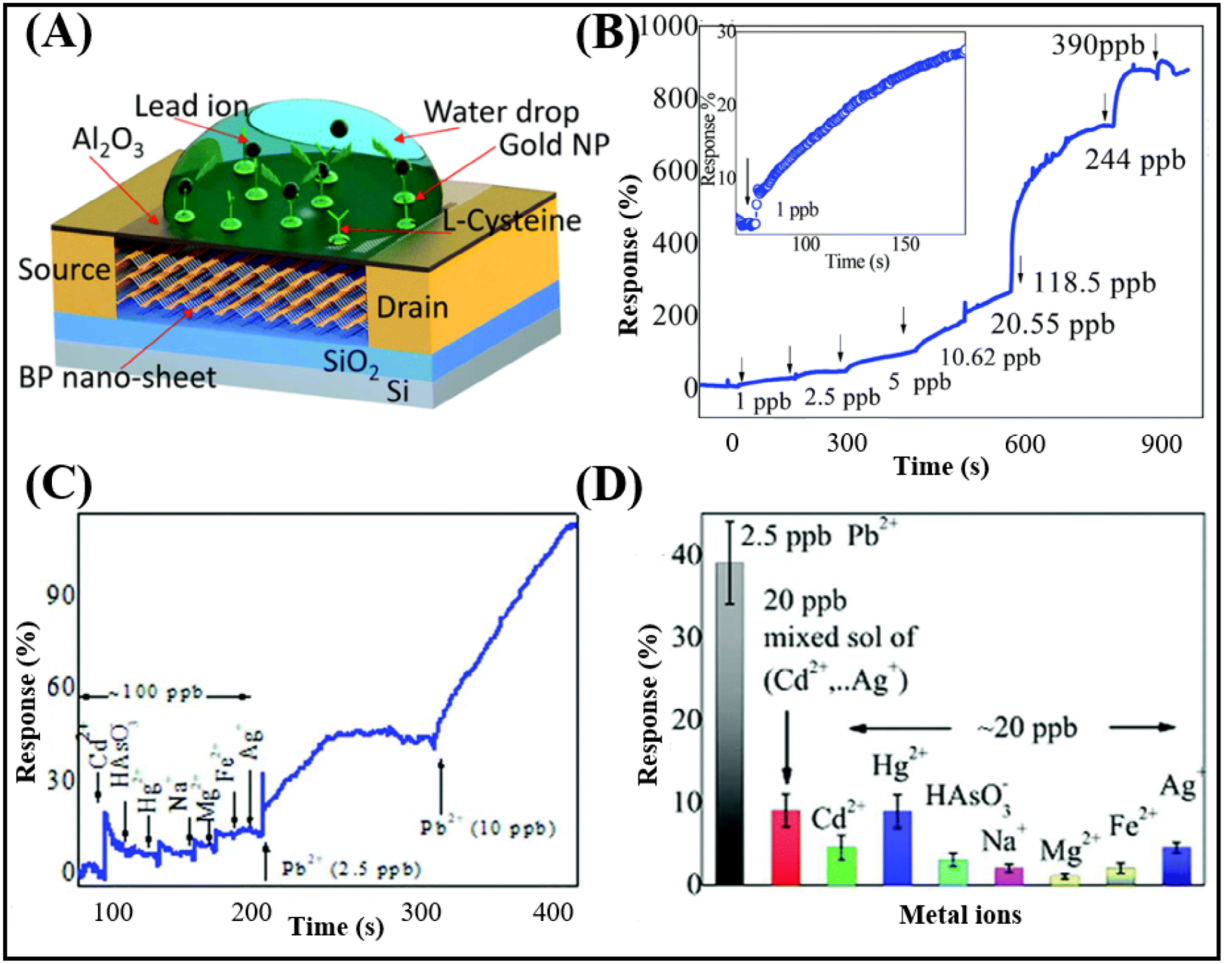
A) Schematic diagram of BP FET modified by cysteine. B) Real‐time response of BP FET detection of different concentrations of Pb^2+^. C) Response diagram of BP FET to Pb^2+^ in a complex solution environment. D) Selectivity of cysteine‐modified BP FET for Pb^2+^.^[^ ^240^ ^]^ Copyright 2020, Royal Society of Chemistry.

**Figure 25:**
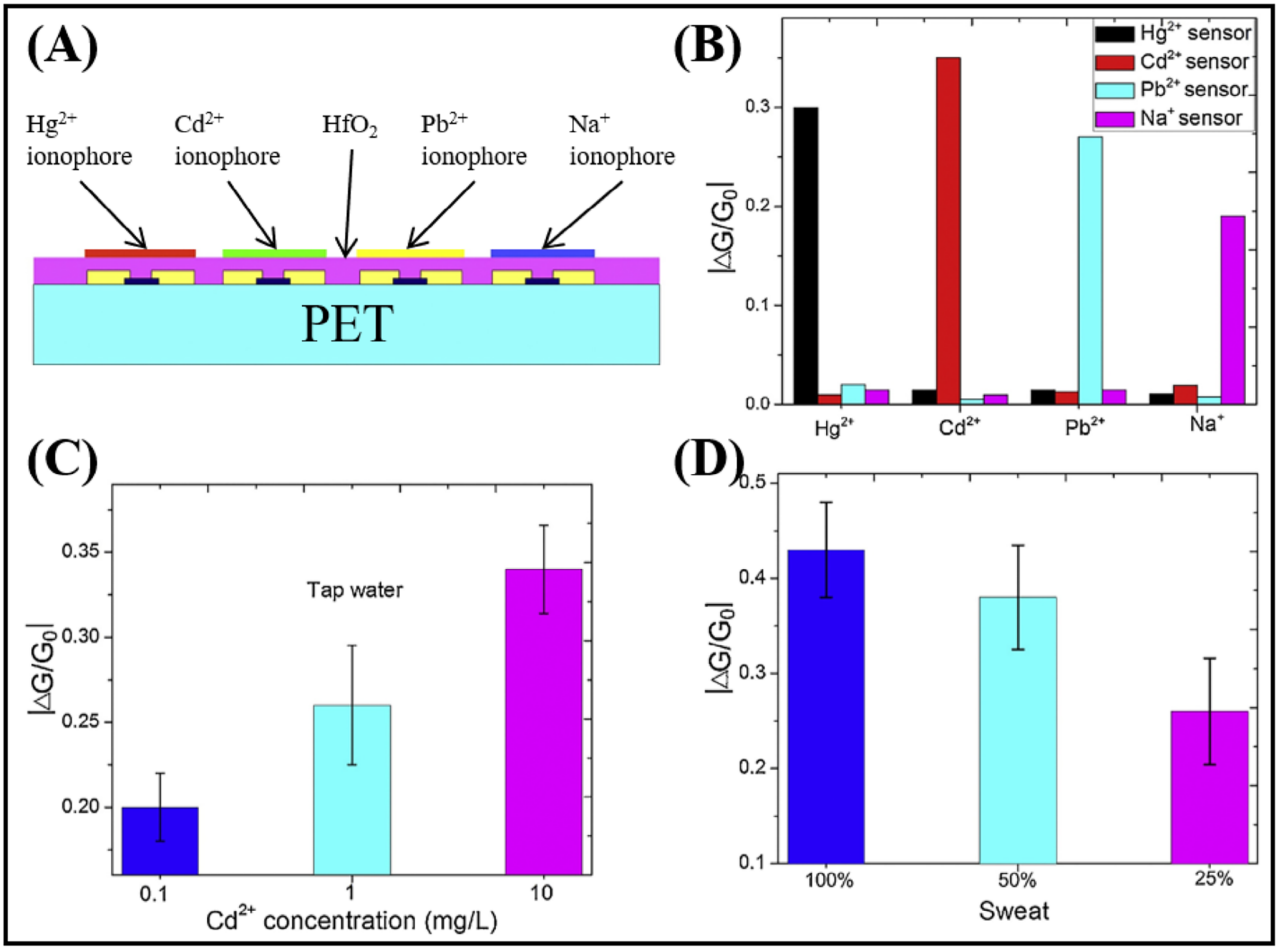
A) Ionophore functionalized FIBA. B) Response of different ionophore modified FIBA to Hg^2+^, Cd^2+^, Pb^2+^, and Na^+^, respectively. C) Cadmium ionophore functionalized FIBA response diagram to tap water with different Cd^2+^ concentrations. D) Response of sodium ionophore functionalized FIBA to different concentrations of sweat.^[^ ^241^ ^]^ Copyright 2018, Elsevier.

Compared to graphene, the direct bandgap of BP inherently determines its sensitivity. When the conductance of the BP FET channel changes, the current variation is larger, leading to stronger current modulation. We summarize the research progress of BP materials in the field of ion detection. Maity et al.^[^ ^240^ ^]^ developed a BP FET for detecting lead ions in water. Due to the instability of BP, a 3 nm Al_2_O_3_ layer was deposited on the surface of BP as a protective layer during device fabrication. Subsequently, cysteine was modified on the Al_2_O_3_ surface, and the ─COOH group in cysteine can specifically bind with lead ions in the solution, thus achieving specific detection of lead ions in a complex solution environment (**Figure** **24**A). The sensor exhibits a LOD as low as 1 ppb, with a response rate of 30% at this concentration. It also shows a response to concentrations up to 390 ppb, with an impressive response rate of 900% (Figure 24B–D). Li and the team^[^ ^241^ ^]^ developed a flexible integrated BP FET sensor arrays (FIBA) capable of detecting multiple ions simultaneously (**Figure** **25**A). In the preparation process of FIBA, the device was first passivated with a 30 nm HfO_2_ layer, followed by functionalization with different ionophores, achieving simultaneous detection of Hg^2+^, Cd^2+^, Pb^2+^, and Na^+^ ions (Figure 25B). Using this FIBA, researchers successfully detected Cd^2+^ ions from tap water samples and observed a significant increase in |Δ*G*/*G*_0_| as the Cd^2+^ concentration increased from 0.1 to 10 mg L^−1^ (Figure 25C). The FIBA was also used to detect Na^+^ ions in human sweat, and when the sweat concentration was reduced from 100% to 25%, the |Δ*G*/*G*_0_| value of the Na^+^ sensor significantly decreased (Figure 25D). These experimental results fully demonstrate the great potential and advantages of BP in the field of ion detection.

Up to now, significant advancements have been made in the detection of ions using Xenes FET sensors, as summarized in **Table** **5** regarding their sensing performance.

**Table 5:** Sensing Performance of Xenes FET Ion Sensors.

Channel material	Analyte	Receptor	LOD	Condition	Refs.
Graphene	Hg^2+^	ionophore	0.1 ppb	ion solutions	[242]
Graphene	Hg^2+^	aptamer	5 nM	1 × PBS	[243]
Graphene	Hg^2+^	ssDNA aptamer	40 pM	Tris‐HCl buffer (0.01 M, pH 7.4)	[244]
Graphene	As^3+^	PBASE	0.02 ppb	back‐gate	[245]
Graphene	As^3+^	DNA probes	5 nM	1 × PBS, pH 7.4	[235]
Graphene	Pb^2+^	DNAzyme probes	1.9 nM	Tris‐HCl buffer (1M, pH 6.8)	[246]
Graphene	Pb^2+^	Pb^2+^‐specific DNAzyme	929.8 fM	HEPES buffer (pH 7.4)	[247]
Graphene	Cu^2+^	thiacalix[4]arene	1µM	Tris‐HCl buffer (20 mM, pH 8.0)	[248]
Graphene	Cu^2+^	functional carbon quantum dots	10 fM	PBS (0.2 mM pH 5.5)	[53]
Graphene	Cu^2+^	aptamer	10 nM	Tris‐HCl buffer (10 mM, pH 8.0)	[249]
Graphene	Cu^2+^	L‐phenylalanine	170 fM	DI water	[250]
Graphene	Ca^2+^	lipid bilayer	50 nM	0.01 × PBS	[65]
Graphene	K^+^	ion selective membrane	<10 µM	DI water	[236]
Graphene	Na^+^	ion selective membrane	<10 µM	DI water	[236]
Graphene	NH_4_^+^	ion selective membrane	<10 µM	DI water	[236]
Graphene	NO_3_^−^	ion selective membrane	<10 µM	DI water	[236]
Graphene	SO_4_^2−^	ion selective membrane	<10 µM	DI water	[236]
Graphene	HPO_4_^2−^	ion selective membrane	<10 µM	DI water	[236]
Graphene	Cl^−^	ion selective membrane	<10 µM	DI water	[236]
Graphene	pH	N/A	2 pH	PBS (10 mM)	[238]
Reduced graphene oxide	Hg^2+^	thioglycolic acid	25 nM	ion solutions	[251]
Reduced graphene oxide	Hg^2+^	DNA probes	1 nM	1 × PBS	[252]
Reduced graphene oxide	pH	poly‐L‐lysine	4 pH	1 × PBS	[239]
Reduced carboxylate graphene oxide	Pb^2+^	lead specific aptamer	0.001 ppb	PBS (pH 7.4)	[253]
BP	Pb^2+^	L‐cysteine	1 ppb	DI water	[240]
BP	Hg^2+^	Hg^2+^ ionophore	10 µg/L	DI water	[241]
BP	Cd^2+^	Cd^2+^ ionophore	1 mg/L	DI water	[241]
BP	Pb^2+^	Pb^2+^ ionophore	1 mg/L	DI water	[241]
BP	Na^+^	Na^+^ ionophore	1 mg/L	DI water	[241]

In summary, Xenes FET biosensors have achieved notable advancements in the detection of various biomarkers and have demonstrated significant advantages. For the FET sensor, the sensing performance mainly depends on the structural design and functionalization of the sensing interface. Therefore, the development of sensors can start from the selection of probes and the functionalization of the sensing interface, to effectively improve the sensing performance of the sensor.

## 5 Conclusion and Future Perspectives

In recent years, FET sensors utilizing single‐element 2D materials have been employed for the detection of various biomarkers, owing to their distinctive structures and superior electronic properties. Compared to conventional optical and electrochemical methods, FET sensors based on single‐element 2D materials exhibit significant advantages, including high sensitivity, high selectivity, and rapid response times. In Section 2, we categorize the device structure based on various sensing interfaces, elaborate on the operational principle of the FET sensor, expound on the impact of Debye length on sensing capabilities, and ultimately present the primary metrics for assessing sensor performance. Sensor systems that employ Xenes FET have been extensively utilized for the detection of proteins, nucleic acids, diminutive biomolecules, and cells. Despite the rapid advancements, there persist several prominent issues concerning Xenes FET biosensors. In this context, we list a few key challenges and discuss the way forward.

### 5.1 Performance Optimization

Currently, FET sensors utilizing single‐element 2D materials are extensively utilized in the realm of biological detection. Among these, graphene is frequently favored due to its distinctive electrical and surface characteristics.^[^ ^85^, ^151^, ^162^ ^]^ Beyond graphene and graphdiyne, BP represents an attractive single‐element 2D material with specific utilities.^[^ ^42^, ^61^ ^]^ Due to distinct material properties, FET biosensors based on different materials exhibit differentiated application priorities. G FET leverage their ultrahigh carrier mobility to enable rapid electron transport and sensitive biomolecule detection.^[^ ^72^ ^]^ However, their zero bandgap results in low on/off ratios that compromise weak electrical signal resolution,^[^ ^41^ ^]^ while surface chemical inertness necessitates complex functionalization to enhance specificity.^[^ ^254^ ^]^ In contrast, BP FET utilize tunable bandgaps to amplify charge transfer effects through energy band matching with biomolecules, thereby improving selectivity. Simultaneously, their high on/off ratios effectively suppress noise interference, enabling stable detection of low‐concentration biomarkers.^[^ ^255^ ^]^ Nevertheless, rapid oxidation in humid/electrolytic environments significantly degrades sensing stability,^[^ ^256^ ^]^ requiring atomic layer deposition encapsulation or surface passivation techniques that increase process complexity and cost.^[^ ^257^ ^]^ Tellurene possesses moderate bandgap, superior environmental stability, high carrier mobility, and ultrahigh on/off ratios. First‐principles calculations reveal monolayer tellurene's nucleobase/aromatic amino acid discrimination mechanism via adsorption energy differences and orbital hybridization‐mediated charge transfer, providing theoretical support for developing high‐sensitivity DNA sequencing chips and targeted biosensors.^[^ ^200^ ^]^ Overall, semimetallic G suits high‐throughput, wide dynamic‐range biosensing applications, whereas semiconductor Xenes like BP and tellurene excel in high‐precision, low‐noise biomolecular detection despite requiring environmental stability optimization.

In addition, germanene^[^ ^258^ ^]^ and arsenene,^[^ ^259^ ^]^ when employed as the foundational material in FETs, exhibit certain applications within the domain of gas sensing, implying that they also have the potential to detect various biological analytes. Moreover, the formation of heterostructures or composites by combining Xenes with other materials presents a promising approach for utilizing them as channel materials in biosensing applications.^[^ ^260^, ^261^ ^]^ Thus, from a material selection perspective, Xenes FET biosensors hold substantial potential and offer a multitude of opportunities for forthcoming developments. Although research on FET biosensing based on single Xenes is rapidly advancing, several challenges in sensing performance remain to be satisfactorily addressed.^[^ ^262^ ^]^ (1) Suboptimal sensitivity in graphene, black phosphorus and tellurene based FET biosensors due to high current noise and excessive carrier concentration. (2) Insufficient specificity caused by nonspecific adsorption of non‐target molecules on hydrophobic Xene surfaces. (3) Poor environmental stability arising from material degradation and thermal noise drift. To address these challenges, the following performance‐enhancing strategies are proposed. ​(1) Heterojunction Interface Engineering: Amplify signals via band structure modulation and optimize carrier mobility through interfacial orbital hybridization, thereby enhancing charge transfer rates, response speed, and sensitivity. (2) ​3D‐Structured Channels: Construct nanopores or corrugated architectures to increase effective sensing area and circumvent Debye screening length limitations for improved sensitivity. ​(3) Opto‐Electro‐Chemical Synergistic Sensing: Develop hybrid mechanisms such as light‐chemical gating in graphene/covalent organic framework (COF) heterostructures to enable photo‐gated detection of small molecules.^[^ ^71^ ^]^ Utilize light‐enhanced surface reactivity in graphene/ZnO heterojunctions for UV‐regulated DNA methylation analysis. (4) ​Surface Functionalization: Directional modification with aptamers or enzymes to enhance target‐specific adsorption, improving selectivity and sensitivity. ​(5) Encapsulation Strategies: Mitigate oxidation, environmental surface defects, and nonspecific adsorption via antifouling coatings or van der Waals encapsulation.

### 5.2 Reusability

Although FET biosensors utilizing Xenes have been extensively adopted, several challenges remain unresolved, including the issue of sensor reusability. Currently, the majority of Xenes FET biosensors are inherently single‐use, primarily due to the difficulty in detaching the receptor layer modified on the sensor interface from the detected analyte post‐detection. Given the relatively high production costs and intricate fabrication processes associated with Xenes FET biosensors, enabling the reuse of individual devices would be of considerable value. To date, the reuse of sensors remains a significant challenge that must be overcome before these sensors can enter the market.

To address the issue of reproducibility, various studies have proposed innovative solutions. For instance, one approach involves integrating channel materials with photocatalysts. This combination facilitates the degradation of organic matter on the material's surface through the photocatalytic activity of reactive oxygen species post‐detection.^[^ ^68^, ^70^ ^]^ Alternatively, detection can be achieved through a reversible reaction where the probe interacts with the target analyte. Subsequently, by altering reaction conditions such as the pH of the environment, the probe can be detached from the analyte, enabling the transistor's reusability.^[^ ^83^ ^]^ The incorporation of magnetic‐responsive aptamers represents a viable strategy for fabricating regeneratable FET biosensing platforms.^[^ ^263^, ^264^ ^]^ Following analyte detection, magnetic field‐mediated detachment of aptamers from Xenes‐based sensors facilitates the regeneration of sensing interfaces while preserving the structural integrity of 2D nanomaterials. Despite these advancements, the development of renewable transistor sensors continues to be a critical and ongoing necessity.

### 5.3 Standardization

The significance of standardization within the sensing domain cannot be overlooked. It encompasses the development of a comprehensive set of standards that underpin the execution of large‐scale production and detection, with the fundamental objective of upholding a high degree of consistency and uniformity throughout all pertinent processes.^[^ ^265^, ^266^ ^]^ Achieving this objective necessitates the utilization of superior quality single‐element 2D materials and the standardization of individual sensor production, thereby diminishing discrepancies among devices. Furthermore, the adoption of standardized manufacturing procedures not only ensures the consistency and dependability of Xenes FET sensor systems but also substantially curtails production expenses.^[^ ^10^ ^]^ This reduction in costs culminates in tangible savings for end consumers, rendering these sophisticated sensing technologies accessible to a broader user base, which in turn bolsters their widespread acceptance and proliferation in the marketplace. Consequently, the standardized manufacturing process confers a substantial economic stimulus for the commercialization of Xenes FET sensors, while concomitantly ensuring their extensive deployment in the healthcare sector and viability within the home testing market.^[^ ^28^ ^]^ In this manner, standardization serves to not only enhance the precision and trustworthiness of the technology but also renders it more user‐friendly, offering a potent instrument for health surveillance and disease diagnosis to users globally.

### 5.4 Combining Biosensors with Artificial Intelligence

Most of the research work mentioned in this review focused on the Xenes FET biosensor developed under controlled experimental conditions. Xenes FET biosensors demonstrate specificity for particular target molecules under ideal experimental conditions.^[^ ^59^, ^82^ ^]^ However, in reality, the biosensor encounters more complex environments, including solutions with intricate compositions.^[^ ^267^ ^]^ Thus, detecting Xenes FET biosensors in complex systems holds significant practical importance. In complex systems, Xenes FET biosensors require substantial data for detection and analysis. Employing artificial intelligence (AI) for data collection, analysis, and prediction is a viable approach. Integrating Xenes FET biosensors with AI and algorithms represents a novel interdisciplinary approach that will play an increasingly important role in the future.

### 5.5 Commercialization

In the current market, despite the presence of several mature biosensing technologies, such as widely used minimally invasive continuous glucose monitoring devices,^[^ ^3^, ^4^, ^268^ ^]^ which continuously track blood sugar fluctuations, Xenes FET biosensors have experienced slower progress in commercial applications, despite demonstrating excellent performance in laboratory studies. Commercial biosensors must satisfy various user requirements, including easy operation, high sensitivity, high reliability, low cost, and real‐time detection. Xenes FET biosensors encounter multiple challenges in their path to commercialization.^[^ ^10^, ^269^, ^270^ ^]^ To promote the commercialization process, addressing stability, ease of use, and scale production, as well as policy support, financial investment, and interdisciplinary cooperation are crucial.

With the continuous progress of science and technology, the prospects for commercializing Xenes FET biosensors are becoming increasingly apparent. Despite these challenges, the technology holds significant future commercial potential owing to its unique advantages. Advances in materials science and micro‐nano processing technology are expected to enable more cost‐effective production methods for large‐area Xenes films, thereby reducing the cost of Xenes FET biosensors and enhancing their market competitiveness.^[^ ^271^, ^272^, ^273^ ^]^ Concurrently, innovations in packaging technology and stabilization treatments will improve the operational stability and environmental adaptability of Xenes FET biosensors, meeting stringent requirements for accuracy and consistency in practical applications.^[^ ^274^, ^275^, ^276^ ^]^ As these technologies mature and the market becomes receptive, Xenes FET biosensors are poised to significantly impact multiple fields and contribute substantially to public health and safety.^[^ ^10^ ^]^

In summary, this paper reviews recent research efforts aimed at elucidating the operating mechanism of Xenes FET in electrolytic environments, as well as the functionalization of 2D material surfaces through specific recognition elements to enhance their sensitivity. We provide an overview of recent advances in protein, nucleic acid, small biomolecules, and cell sensors that utilize single‐element 2D materials as sensing elements. Given the substantial research efforts in this field, we anticipate that Xenes FET biosensors will soon overcome challenges such as Debye length limitations, regenerability, and commercialization. Further expanding the possibilities for biomolecule detection is the integration of AI with Xenes FET biosensors, which further propels their development toward miniaturization, scalability, low power consumption, cost‐effectiveness, and high sensitivity.

## Conflict of Interest

The authors declare no conflict of interest.
